# Unraveling indications for discharge antibiotics: the Devil’s in the details

**DOI:** 10.1017/ash.2025.10123

**Published:** 2025-09-22

**Authors:** Ritika Prasad, Marten R. Hawkins, Radhika Arya, Alex N. Zimmet, Leah Mische, Emily Mui, Esther Esadah, Samaneh Pourali, David R. Ha, Marisa Holubar

**Affiliations:** 1 Division of Infectious Diseases and Geographic Medicine, Department of Medicine, Stanford University School of Medicine, Stanford, CA, USA; 2 Department of Quality, Safety & Health Equity, Stanford University School of Medicine, Stanford, CA, USA; 3 Division of Infectious Diseases, Department of Medicine, University of Michigan Medical School, Ann Arbor, MI, USA; 4 Division of Infectious Diseases, Department of Medicine, Medical College of Wisconsin, Milwaukee, WI, USA; 5 Children’s National Hospital, Washington, DC, USA; 6 Department of Pharmacy, Stanford Health Care, Stanford, CA, USA

## Abstract

**Objective:**

Discharge is a pivotal transition of care moment; however, discharge antibiotic stewardship efforts have historically been limited. Our institution has a mandatory indication field for inpatient antibiotic orders. Using the last-ordered inpatient antibiotic indication (“Last Inpatient Indication”), we assessed the utility of this field compared to the ICD-10 in inferring clinician intent for discharge antibiotics, to forgo the need for manual chart review to assess antibiotic appropriateness.

**Methods:**

We extracted electronic medical record data on adult inpatient encounters in 2023 with ≤2 discharge antibiotics. We reviewed a random subset of 300 encounters to determine if the ICD-10 or Last Inpatient Indication had higher agreement with clinician intent for discharge antibiotics, as determined by chart notes. To facilitate comparison, we created a dictionary classifying ICD-10 indications.

**Results:**

We included 3,414 encounters. The most common discharge antibiotics were amoxicillin/clavulanate (24%) and ciprofloxacin (15%). The most common ICD-10s were non-infectious (48%) and sepsis (18%). In the subset of chart-reviewed encounters, the Last Inpatient Indication agreed with the documented clinician intent for discharge antibiotic more often than the ICD-10 (84% vs 28%). Applying institutional guidelines, we were able to use the Last Inpatient Indication to assess appropriate duration and choice of discharge antibiotics for select infections.

**Conclusions:**

The Last Inpatient Indication outperformed ICD-10s in inferring clinician intent for discharge antibiotics and can be used to efficiently assess antibiotic appropriateness without need for manual chart review. Usefulness of ICD-10s was limited by high percentage of non-infectious and sepsis codes.

## Introduction

The time of discharge is a pivotal point in a patient’s care when key antibiotic decisions are made.^1^ Nearly one in six admissions results in a discharge antibiotic prescription, and up to 70% of antibiotic prescribing at that time could be further optimized.^2–4^ The stakes of discharge antibiotic prescriptions are high because monitoring patients in the outpatient setting for antibiotic adherence, side effects, and infection relapse can be challenging and resource-intensive. With hospitals increasingly focused on reducing length of stay as well as readmissions, optimizing efficient and safe discharges with appropriate antibiotics presents an opportunity for antibiotic stewardship programs (ASPs) to directly support these priorities and secure needed resources for this goal.

Previous ASP efforts to optimize discharge antibiotics have largely used manual chart review to identify opportunities, a process that is unsustainable and impractical for increasingly complex and large healthcare systems as well as for smaller hospitals that may have disproportionately fewer ASP resources.^3,5^ A method leveraging electronic medical record (EMR) data to accurately assess indications for discharge antibiotics could help ASPs efficiently review large volumes of discharge antibiotics and identify targets for intervention. The International Classification of Diseases, Tenth Revision (ICD-10) code has previously been used to infer the reasons why antibiotics are prescribed but its concordance with clinician intent remains unclear, with limited data on its use in outpatient and discharge settings.^6–8^

Some institutions have adopted a mandatory indication field in electronic antibiotic orders, which could serve as an alternative to the ICD-10 to infer syndrome for which an antibiotic is prescribed.^8^ At our institution, only inpatient antibiotic orders contain a mandatory indication field; discharge and outpatient antibiotic orders do not. We aimed to assess whether the last manually entered inpatient antibiotic order indication, hereafter referred to as Last Inpatient Indication, is more accurate than the ICD-10 for inferring the clinician-intended discharge antibiotic indication. Using the Last Inpatient Indication, we also piloted a method to assess guideline concordance of antibiotics prescribed at discharge.

## Methods

### Design and data collection

**Figure 1.**
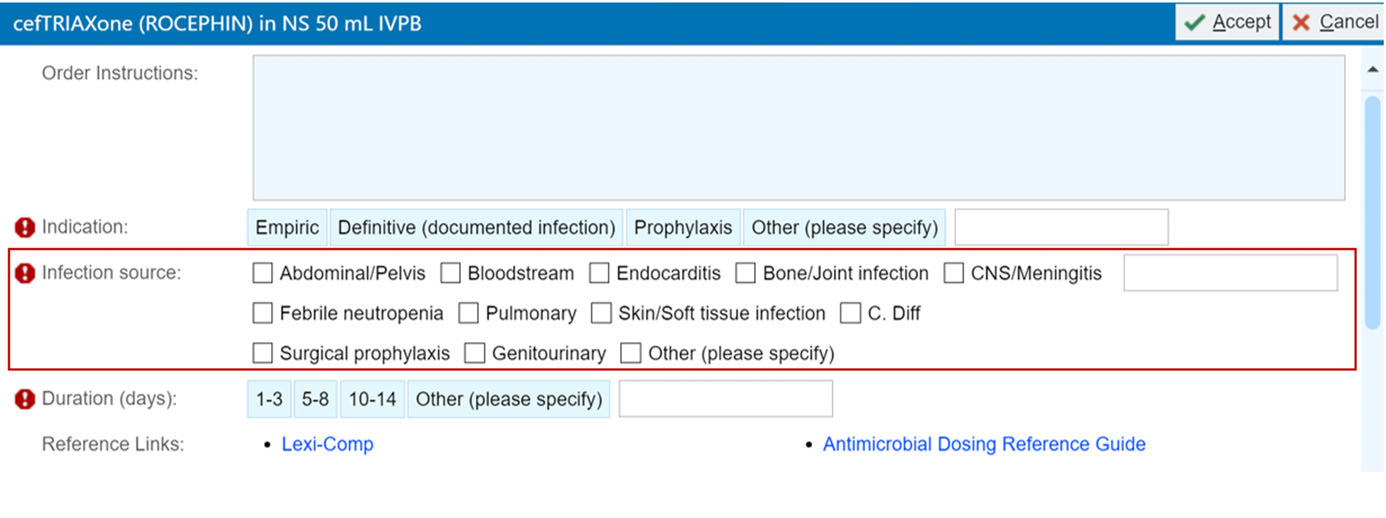
Image capture of inpatient antibiotic order with mandatory indication field outlined in a red box.

In this single-center retrospective project, we included all adult inpatient encounters at our institution associated with ≤2 oral antibiotics upon discharge between January and December 2023. From the EMR, we extracted provider type, team type, discharge antibiotic names, and encounter billing ICD-10 codes. Our institution has a mandatory indication field for all inpatient antibiotics and includes checkboxes for common inpatient infectious sources as well as an “Other” free-text field (Figure 1). From the EMR, we extracted the Last Inpatient Indication, defined as the indication field for the last inpatient antibiotic ordered in the encounter. We excluded encounters in which the Last Inpatient Indication was “Surgical prophylaxis” or “Other.” We excluded encounters with >2 discharge antibiotics to facilitate straightforward comparison between documented discharge antibiotic indication, Last Inpatient Indication and ICD-10. We excluded discharge antibiotics with duration >90 days, since these antibiotics were often given for hematologic or oncologic prophylaxis. Durations ≤90 days were expected to include most treatment courses for common infections, including those requiring prolonged therapy such as endocarditis. We excluded encounters associated with discharge antivirals, antifungals, antimycobacterials, and antibiotics prescribed via intramuscular or intravenous formulations. Parenteral discharge antibiotics were excluded because they typically do not involve an electronic antibiotic order but a communication to a receiving nursing facility or home health agency that is more difficult to track.

To facilitate comparison of ICD-10 with Last Inpatient Indication, we created a data dictionary classifying ICD-10s (Supplementary Table 1) from our database of included patient encounters into diagnosis categories that aligned with Last Inpatient Indication options (Figure 1). We also classified ICD-10s into a sepsis category if they coded for a sepsis etiology without a specific infectious source. ICD-10s without an infectious etiology were classified as non-infectious. ICD-10s with infectious etiologies that do not require antibiotics (eg, viral infections) were also classified as non-infectious since they were not relevant to discharge antibiotic indication.

We randomly selected 300 encounters (8–10% of the estimated 3,500 encounters available) to manually chart-review the discharge antibiotic indication documented in discharge summaries and, when applicable, infectious diseases consultant notes. Up to three clinician-intended indications could be assigned to a discharge antibiotic since some antibiotics were prescribed for multiple infectious categories (e.g., documentation describes an antibiotic that was prescribed for bacteremia due to urinary tract infection (UTI), as well as pneumonia). This protocol was deemed to be non-human subjects research by Stanford’s Institutional Review Board.

### Analysis

We used descriptive statistics to summarize ordering provider type, team group, and Last Inpatient Indications. In the chart-reviewed subset of encounters, we determined agreement of ICD-10 and Last Inpatient Indication with documented discharge antibiotic indication as percentage that matched. If the ICD-10 category was sepsis, we did not consider it as a match with the Last Inpatient Indication or documented discharge antibiotic indication because sepsis ICD-10s were not specific enough to draw conclusions regarding source of infection and thus assess appropriateness of discharge antibiotics.

Finally, for all encounters, we used the Last Inpatient Indication to identify the most extreme cases of guideline discordance for common infections compared to institutional guidelines. We reported the proportion of encounters that were guideline concordant with respect to duration and antibiotic choice. Guideline-concordant antibiotic choices were defined by institutional guidelines and are listed in Supplementary Table 2. We defined the following guideline-concordant durations of discharge antibiotic prescriptions: ≤7 days for pulmonary infections, ≤7 days for genitourinary infections, and ≤10 days for *C. difficile* infections. These durations were chosen to reflect institutional guideline-recommended durations of up to 5 days for community-acquired pneumonia, up to 7 days for hospital-acquired or ventilator-associated pneumonia, 3–5 days for UTI, 7 days for complicated UTI, and 10 days for first case of *C. difficile* infection. The criteria for guideline-concordant durations relied only on the duration of the discharge antibiotic prescription and did not account for inpatient duration of therapy; these criteria were intentionally permissive to be reproducible, practical without need for extensive chart review, and to prioritize identification of the most extreme durations.

## Results

A total of 3,414 encounters met our inclusion criteria. The most common discharge antibiotics were amoxicillin/clavulanate (24%) and ciprofloxacin (15%). Seventeen percent of encounters had more than one discharge antibiotic (Table 1). The most common indications for the Last Inpatient Indication were genitourinary (23%) and pulmonary (22%) (Table 1). In contrast, the most common diagnoses for ICD-10s were non-infectious (48%) and sepsis (18%), while genitourinary infectious codes only made up 7% of ICD-10s.

**Table 1.** Baseline characteristics of all encounters with discharge antibiotics and chart-reviewed encounters^a^

	All encounters (*n* = 3,414)	Chart-reviewed encounters (*n* = 300)
Median length of stay—days	4.3	4.7
Discharged with two antibiotics—*n* (%)	583 (17%)	53 (18%)
Team group—*n* (%)		
Medicine	1,440 (42%)	115 (38%)
Surgery	1,040 (30%)	86 (29%)
Medicine subspecialty^b^	934 (27%)	99 (33%)
Provider type—*n* (%)		
Trainee	827 (24%)	78 (26%)
Advanced practice provider	1,085 (32%)	94 (31%)
Attending	1502 (44%)	128 (43%)
Last inpatient indication—*n* (%)		
Abdomen/pelvis	689 (20%)	60 (20%)
Bloodstream	232 (7%)	19 (6%)
Endocarditis	7 (0%)	0 (0%)
Bone and Joint	112 (3%)	5 (2%)
*C. difficile*	61 (2%)	8 (3%)
CNS/meningitis	11 (0%)	1 (0%)
Febrile neutropenia	60 (2%)	8 (3%)
Genitourinary	783 (23%)	71 (24%)
Pulmonary	740 (22%)	55 (18%)
Skin and soft tissue	719 (21%)	73 (24%)

a Percentages may not total 100 because of rounding.

b Medicine subspecialty teams included Hematology, Oncology, Gastroenterology, and other Internal Medicine subspecialties that have admitting teams at our institution.

In the subset of chart-reviewed encounters, the most common Last Inpatient Indications were genitourinary (24%) and skin and soft tissue (24%), which were also the most common documented discharge antibiotic indications, while the most common ICD-10 diagnoses were non-infectious (52%) (Table 2). The ICD-10 source-specific infectious diagnoses of abdomen/pelvis, bone and joint, CNS (central nervous system)/meningitis, genitourinary, pulmonary, and skin and soft tissue had >90% agreement with documented discharge antibiotic indications. However, only 28% of all ICD-10s agreed with the documented discharge antibiotic indications (Table 3), with low overall agreement driven by non-infectious and sepsis ICD-10s. In contrast, 84% of Last Inpatient Indications agreed with documented discharge antibiotic indication (Table 3). The Last Inpatient Indications with the highest agreement with documented discharge antibiotic indications were CNS/meningitis (100%), bone and joint (100%), genitourinary (94%), *C. difficile* (88%), and pulmonary (87%), while febrile neutropenia had the lowest agreement (25%) (Table 4). Although we excluded encounters in which the Last Inpatient Indication was surgical prophylaxis, 2% of the chart-reviewed encounters had discharge antibiotics prescribed for prophylaxis per documentation. Another 2% of chart-reviewed encounters had discharge antibiotics without a clearly documented indication.

**Table 2.** Encounters categorized into Last Inpatient Indications, ICD-10 categories, and documented discharge antibiotic indication categories in chart-reviewed encounters (N = 300)^a^

	Last Inpatient Indication—*n* (%)	ICD-10^b^—*n* (%)	Documented Discharge Antibiotic Indication^b^—*n* (%)
Abdomen/pelvis	60 (20%)	21 (7%)	57 (19%)
Bloodstream	19 (6%)	3 (1%)	30 (10%)
Endocarditis	0 (0%)	0 (0%)	0 (0%)
Bone and Joint	5 (2%)	3 (1%)	13 (4%)
*C. difficile*	8 (3%)	2 (1%)	8 (3%)
CNS/meningitis	1 (0%)	1 (0%)	1 (0%)
Febrile neutropenia	8 (3%)	0 (0%)	4 (1%)
Genitourinary	71 (24%)	13 (4%)	79 (26%)
Pulmonary	55 (18%)	17 (6%)	53 (18%)
Skin and soft tissue	73 (24%)	27 (9%)	68 (23%)
Prophylaxis	–	–	14 (5%)
Sepsis^c^	–	51 (17%)	–
Non-infectious	–	157 (52%)	–
No clinician intent documented	–	–	5 (2%)

a Percentages may not total 100 due to rounding.

b Percentages may not total 100 since each encounter could have up to two discharge antibiotics, and up to three clinician intent indications could be assigned to each antibiotic. Encounters with indication of “Other” are not displayed in this table.

c ICD-10 sepsis codes were not counted as matches with any Last Inpatient Indication or documented discharge antibiotic indication.

**Table 3.** Agreement of ICD-10 code vs. Last Inpatient Indication with documented discharge antibiotic indication in chart-reviewed encounters (*n* = 300)

	ICD-10 code—*n* (%)	Last Inpatient Indication—*n* (%)
Discharge antibiotic agreed with documented indication	84 (28%)	252 (84%)
Discharge antibiotic disagreed with documented indication	216 (72%)	48 (16%)

**Table 4.** Agreement of Last Inpatient Indication with documented indication for discharge antibiotic in chart-reviewed encounters (N = 300)

Last Inpatient Indication	Agreement of discharge antibiotic documented indication—*n* (%)	Disagreement of discharge antibiotic with documented indication—*n* (%)
Abdomen/pelvis	46/60 (77%)	14/60 (23%)
Bloodstream	14/19 (74%)	5/19 (26%)
Endocarditis^a^	–	–
Bone and Joint	5/5 (100%)	0/5 (0%)
*C. difficile*	7/8 (88%)	1/8 (13%)
CNS/meningitis	1/1 (100%)	0/1 (0%)
Febrile neutropenia	2/8 (25%)	6/8 (75%)
Genitourinary	67/71 (94%)	4/71 (6%)
Pulmonary	48/55 (87%)	7/55 (13%)
Skin and soft tissue	62/73 (85%)	11/73 (15%)

a There were no encounters in the 300 manually reviewed encounters in which the Last Inpatient Indication was endocarditis.

In the entire data set of 3,414 encounters, the encounters with pulmonary, *C. difficile*, and genitourinary Last Inpatient Indications had high guideline-concordant discharge antibiotic choices and durations of >75% (Table 5).

**Table 5.** Guideline concordance of discharge antibiotics in encounters categorized by Last Inpatient Indication for selected infections

Last Inpatient Indication	Guideline concordance of discharge antibiotic choice^a^—*n* (%)	Guideline concordance of discharge antibiotic duration^b^—*n* (%)
Pulmonary	751/873 (86%)	720/873 (82%)
*C. difficile*	59/69 (86%)	53/69 (77%)
Genitourinary	678/824 (82%)	658/824 (80%)

a Discharge antibiotic choice was considered guideline-concordant if it was in line with institutional guidelines for pulmonary, genitourinary, and *C. difficile* indications (Supplementary Table 2).

b Discharge antibiotic duration was considered guideline-concordant if it was ≤7 days for pulmonary, ≤7 days for genitourinary, and ≤10 days for *C. difficile* indications.

## Discussion

We found that the Last Inpatient Indication had a higher agreement with documented discharge antibiotic indication compared to ICD-10s. This suggests that extracting the Last Inpatient Indication from the EMR could be an efficient and accurate way for ASPs to infer clinician intent for discharge antibiotics without manual chart review, especially in institutions where inpatient antibiotic orders have an indication field but discharge antibiotic orders do not. Prior work has been consistent with our findings, showing that inpatient mandatory indication fields are more accurate than ICD-10s in reflecting documented antibiotic indications, and that there is high agreement between mandatory indication fields and documented indications, ranging from 74% to 95% in the inpatient setting and 93% in the outpatient setting.^8–12^ However, few studies have examined the discharge context; our work extends the literature by comparing the accuracy of inpatient mandatory indication fields and ICD-10s with regards to discharge antibiotic indications.

Although ICD-10s are widely available, our project highlights that they may be flawed in assessing indications for discharge antibiotics. Nearly half of ICD-10s (48%) in our encounter data set coded non-infectious diagnoses, suggesting that discharge antibiotics are not always related to the ICD-10 used for encounter billing purposes. Among ICD-10 infectious categories, sepsis was the most common (18%); however, sepsis codes were not source-specific and provided little insight on appropriate antibiotic choice, dose or duration.

The minority of ICD-10s that did code for a non-sepsis infectious etiology had high agreement with clinician intent for discharge antibiotics. However, because most ICD-10s at our center were either non-infectious or sepsis-related, overall they offered limited utility for ASP reviewers in assessing antibiotic appropriateness without labor-intensive and unsustainable chart review. Others have used ICD-10s to infer reasons for antibiotic prescriptions, but reported concordance with documented indications varies widely in the literature, ranging from 32–95% in inpatient settings.^6–8^ This variability suggests that institutional culture and EMR-related factors may affect the accuracy of ICD-10s in capturing discharge antibiotic indications. Therefore, an ICD-10-based approach to ASP review should be used with caution and not assumed to be best practice without validation.

ASPs are best served by reliable, actionable, and reproducible metrics that are sustainable long-term. Although previous studies have leveraged the EMR to assess discharge antibiotic appropriateness, these efforts have largely relied on manual chart review to ascertain indication, appropriate duration and choice.^13,14^ Chart review is useful for validation and investigational purposes but usually cannot be relied upon as a long-term data source given its time-consuming nature. One strength of our method is that it would reduce the need for extensive manual chart review by limiting chart review to cases in which antibiotics were identified as guideline-discordant. Another strength of this method is its applicability across all hospital service lines and many infectious syndromes. ASPs could identify specific infectious syndromes or hospital service lines with low rates of guideline-concordant discharge antibiotics and provide prescribers with feedback as a performance metric.

Our work suggests that some inpatient antibiotic order indications may be more useful than others in inferring documented indication for discharge antibiotics. Most Last Inpatient Indications were >70% concordant with documented discharge antibiotic indications, except for febrile neutropenia, which had only 25% concordance. This suggests that febrile neutropenia may be an initial empiric diagnosis more relevant early in hospitalization, with a specific source identified later on, limiting its relevance to discharge antibiotic indication.

Our project has several limitations. First, our findings may not be generalizable to all institutions due to variability in EMR, ICD-10 coding, and antibiotic prescribing practices. Second, given the retrospective nature of this project, our findings are limited by available documentation and may not fully capture clinical decision-making. Finally, our definition of guideline-concordant discharge antibiotic duration was based solely on the duration of the discharge antibiotic and did not account for the duration of inpatient antibiotics that could have contributed to the overall length of therapy. This permissive approach was chosen to reflect a practical approach for ASPs starting work in this area, but in doing so we may have overestimated guideline concordance of durations. We also did not have access to patient characteristics to determine if some patients would qualify for different durations than outlined in our guideline concordance criteria. More work is needed to account for both inpatient and discharge antibiotic duration before this level of data is actionable.

In conclusion, we demonstrated that the Last Inpatient Indication performed better than ICD-10s in aligning with documented discharge antibiotic indication, making it a valuable tool for assessing discharge antibiotics. Institutions that have a mandatory indication field on inpatient antibiotic orders, but not for discharge antibiotics, may find the Last Inpatient Indication useful for ASP review of discharge antibiotic guideline concordance. Institutions may also find it pragmatic to assess concordance with their institutional guidelines on a large scale by creating criteria for guideline concordance and applying these criteria using the Last Inpatient Indication to assess appropriateness of discharge antibiotics. Further work is needed to assess best practices for creating and validating these guideline concordance criteria. Given the wide variability in reported agreement between ICD-10s and antibiotic indications in the literature, further review of ICD-10 distribution at other centers may be valuable.

## Supporting information

10.1017/ash.2025.10123.sm001 Prasad et al. supplementary material 1 Prasad et al. supplementary material

10.1017/ash.2025.10123.sm002 Prasad et al. supplementary material 2 Prasad et al. supplementary material

## Supplementary material

The supplementary material for this article can be found at https://doi.org/10.1017/ash.2025.10123.
