# Supplementary material for: Unraveling indications for discharge antibiotics: the Devil’s in the details
Source: Antimicrob Steward Healthc Epidemiol. 2025 Sep 22;5(1):e233. doi: 10.1017/ash.2025.10123 (PMC12509150; doi:10.1017/ash.2025.10123)
Supplement: Prasad et al. supplementary material 1 — Prasad et al. supplementary material [file S2732494X2510123Xsup001.docx]

**Supplementary Table 1.** ICD-10 dictionary classifying encounter billing ICD-10 codes into syndromic infection categories. ICD-10 dictionary is derived from the 3,414 encounters included in the study. Infections that were non-bacterial or without indication for antibiotics were categorized as non-infectious (e.g. viral infections).

| ICD-10 | ICD-10 Description | ICD-10 Category |
| --- | --- | --- |
| A02.0 | Salmonella enteritis | Abdomen/pelvis |
| A02.1 | Salmonella sepsis | Sepsis |
| A03.9 | Shigellosis, unspecified | Abdomen/pelvis |
| A04.4 | Other intestinal Escherichia coli infections | Abdomen/pelvis |
| A04.5 | Campylobacter enteritis | Abdomen/pelvis |
| A04.6 | Enteritis due to Yersinia enterocolitica | Abdomen/pelvis |
| A04.71 | Enterocolitis due to Clostridium difficile, recurrent | C. difficile |
| A04.72 | Enterocolitis due to Clostridium difficile, not specified as recurrent | C. difficile |
| A04.8 | Other specified bacterial intestinal infections | Abdomen/pelvis |
| A04.9 | Bacterial intestinal infection, unspecified | Abdomen/pelvis |
| A05.8 | Other specified bacterial foodborne intoxications | Non-infectious |
| A05.9 | Bacterial foodborne intoxication, unspecified | Non-infectious |
| A06.0 | Acute amebic dysentery | Non-infectious |
| A06.4 | Amebic liver abscess | Non-infectious |
| A07.1 | Giardiasis (lambliasis) | Non-infectious |
| A07.2 | Cryptosporidiosis | Non-infectious |
| A08.11 | Acute gastroenteropathy due to Norwalk agent | Non-infectious |
| A08.39 | Other viral enteritis | Non-infectious |
| A08.4 | Viral intestinal infection, unspecified | Non-infectious |
| A09 | Infectious gastroenteritis and colitis, unspecified | Abdomen/pelvis |
| A15.0 | Tuberculosis of lung | Non-infectious |
| A19.8 | Other miliary tuberculosis | Non-infectious |
| A19.9 | Miliary tuberculosis, unspecified | Non-infectious |
| A23.8 | Other brucellosis | Other |
| A28.0 | Pasteurellosis | Other |
| A31.0 | Pulmonary mycobacterial infection | Pulmonary |
| A31.2 | Disseminated mycobacterium avium-intracellulare complex (DMAC) | Other |
| A39.0 | Meningococcal meningitis | CNS/meningitis |
| A40.0 | Sepsis due to Streptococcus, group A | Sepsis |
| A40.1 | Sepsis due to Streptococcus, group B | Sepsis |
| A40.3 | Sepsis due to Streptococcus pneumoniae | Sepsis |
| A40.8 | Other streptococcal sepsis | Sepsis |
| A40.9 | Streptococcal sepsis, unspecified | Sepsis |
| A41.01 | Sepsis due to methicillin susceptible Staphylococcus aureus | Sepsis |
| A41.02 | Sepsis due to methicillin resistant Staphylococcus aureus | Sepsis |
| A41.1 | Sepsis due to other specified Staphylococcus | Sepsis |
| A41.2 | Sepsis due to unspecified Staphylococcus | Sepsis |
| A41.3 | Sepsis due to hemophilus influenzae | Sepsis |
| A41.4 | Sepsis due to anaerobes | Sepsis |
| A41.50 | Gram-negative sepsis, unspecified | Sepsis |
| A41.51 | Sepsis due to Escherichia coli (e. coli) | Sepsis |
| A41.52 | Sepsis due to Pseudomonas | Sepsis |
| A41.53 | Sepsis due to Serratia | Sepsis |
| A41.54 | Sepsis due to Acinetobacter baumannii | Sepsis |
| A41.59 | Other gram-negative sepsis | Sepsis |
| A41.81 | Sepsis due to Enterococcus | Sepsis |
| A41.89 | Other specified sepsis | Sepsis |
| A41.9 | Sepsis, unspecified organism | Sepsis |
| A46 | Erysipelas | SSTI |
| A48.1 | Legionnaires' disease | Pulmonary |
| A51.39 | Other secondary syphilis of skin | SSTI |
| A52.3 | Neurosyphilis, unspecified | CNS/meningitis |
| A54.24 | Gonococcal female pelvic inflammatory disease | Genitourinary |
| A54.86 | Gonococcal sepsis | Sepsis |
| A54.89 | Other gonococcal infections | Genitourinary |
| A56.11 | Chlamydial female pelvic inflammatory disease | Genitourinary |
| A68.1 | Tick-borne relapsing fever | Other |
| A69.20 | Lyme disease, unspecified | Other |
| A69.22 | Other neurologic disorders in Lyme disease | Other |
| A78 | Q fever | Other |
| B00.1 | Herpesviral vesicular dermatitis | Non-infectious |
| B00.7 | Disseminated herpesviral disease | Non-infectious |
| B00.9 | Herpesviral infection, unspecified | Non-infectious |
| B02.0 | Zoster encephalitis | Non-infectious |
| B02.29 | Other postherpetic nervous system involvement | Non-infectious |
| B02.30 | Zoster ocular disease, unspecified | Non-infectious |
| B02.39 | Other herpes zoster eye disease | Non-infectious |
| B02.7 | Disseminated zoster | Non-infectious |
| B02.8 | Zoster with other complications | Non-infectious |
| B20 | Human immunodeficiency virus (HIV) disease | Non-infectious |
| B25.0 | Cytomegaloviral pneumonitis | Non-infectious |
| B34.8 | Other viral infections of unspecified site | Non-infectious |
| B37.0 | Candidal stomatitis | Non-infectious |
| B37.49 | Other urogenital candidiasis | Non-infectious |
| B37.7 | Candidal sepsis | Non-infectious |
| B38.2 | Pulmonary coccidioidomycosis, unspecified | Non-infectious |
| B44.0 | Invasive pulmonary aspergillosis | Non-infectious |
| B44.1 | Other pulmonary aspergillosis | Non-infectious |
| B44.81 | Allergic bronchopulmonary aspergillosis | Non-infectious |
| B45.0 | Pulmonary cryptococcosis | Non-infectious |
| B46.0 | Pulmonary mucormycosis | Non-infectious |
| B46.1 | Rhinocerebral mucormycosis | Non-infectious |
| B48.8 | Other specified mycoses | Non-infectious |
| B59 | Pneumocystosis | Non-infectious |
| B69.0 | Cysticercosis of central nervous system | Non-infectious |
| B81.8 | Other specified intestinal helminthiases | Non-infectious |
| B86 | Scabies | Non-infectious |
| B99.9 | Unspecified infectious disease | Other |
| C00.1 | Malignant neoplasm of external lower lip | Non-infectious |
| C01 | Malignant neoplasm of base of tongue | Non-infectious |
| C02.1 | Malignant neoplasm of border of tongue | Non-infectious |
| C02.2 | Malignant neoplasm of ventral surface of tongue | Non-infectious |
| C02.8 | Malignant neoplasm of overlapping sites of tongue | Non-infectious |
| C02.9 | Malignant neoplasm of tongue, unspecified | Non-infectious |
| C04.8 | Malignant neoplasm of overlapping sites of floor of mouth | Non-infectious |
| C05.0 | Malignant neoplasm of hard palate | Non-infectious |
| C06.2 | Malignant neoplasm of retromolar area | Non-infectious |
| C06.9 | Malignant neoplasm of mouth, unspecified | Non-infectious |
| C07 | Malignant neoplasm of parotid gland | Non-infectious |
| C08.1 | Malignant neoplasm of sublingual gland | Non-infectious |
| C08.9 | Malignant neoplasm of major salivary gland, unspecified | Non-infectious |
| C09.0 | Malignant neoplasm of tonsillar fossa | Non-infectious |
| C09.8 | Malignant neoplasm of overlapping sites of tonsil | Non-infectious |
| C09.9 | Malignant neoplasm of tonsil, unspecified | Non-infectious |
| C11.8 | Malignant neoplasm of overlapping sites of nasopharynx | Non-infectious |
| C11.9 | Malignant neoplasm of nasopharynx, unspecified | Non-infectious |
| C13.9 | Malignant neoplasm of hypopharynx, unspecified | Non-infectious |
| C14.0 | Malignant neoplasm of pharynx, unspecified | Non-infectious |
| C14.8 | Malignant neoplasm of overlapping sites of lip, oral cavity and pharynx | Non-infectious |
| C15.3 | Malignant neoplasm of upper third of esophagus | Non-infectious |
| C15.4 | Malignant neoplasm of middle third of esophagus | Non-infectious |
| C15.5 | Malignant neoplasm of lower third of esophagus | Non-infectious |
| C15.8 | Malignant neoplasm of overlapping sites of esophagus | Non-infectious |
| C15.9 | Malignant neoplasm of esophagus, unspecified | Non-infectious |
| C16.0 | Malignant neoplasm of cardia | Non-infectious |
| C16.2 | Malignant neoplasm of body of stomach | Non-infectious |
| C16.3 | Malignant neoplasm of pyloric antrum | Non-infectious |
| C16.5 | Malignant neoplasm of lesser curvature of stomach, unspecified | Non-infectious |
| C16.8 | Malignant neoplasm of overlapping sites of stomach | Non-infectious |
| C16.9 | Malignant neoplasm of stomach, unspecified | Non-infectious |
| C17.0 | Malignant neoplasm of duodenum | Non-infectious |
| C17.2 | Malignant neoplasm of ileum | Non-infectious |
| C17.8 | Malignant neoplasm of overlapping sites of small intestine | Non-infectious |
| C17.9 | Malignant neoplasm of small intestine, unspecified | Non-infectious |
| C18.0 | Malignant neoplasm of cecum | Non-infectious |
| C18.1 | Malignant neoplasm of appendix | Non-infectious |
| C18.2 | Malignant neoplasm of ascending colon | Non-infectious |
| C18.3 | Malignant neoplasm of hepatic flexure | Non-infectious |
| C18.4 | Malignant neoplasm of transverse colon | Non-infectious |
| C18.5 | Malignant neoplasm of splenic flexure | Non-infectious |
| C18.6 | Malignant neoplasm of descending colon | Non-infectious |
| C18.7 | Malignant neoplasm of sigmoid colon | Non-infectious |
| C19 | Malignant neoplasm of rectosigmoid junction | Non-infectious |
| C20 | Malignant neoplasm of rectum | Non-infectious |
| C22.0 | Liver cell carcinoma | Non-infectious |
| C22.1 | Intrahepatic bile duct carcinoma | Non-infectious |
| C22.9 | Malignant neoplasm of liver, not specified as primary or secondary | Non-infectious |
| C23 | Malignant neoplasm of gallbladder | Non-infectious |
| C24.0 | Malignant neoplasm of extrahepatic bile duct | Non-infectious |
| C24.1 | Malignant neoplasm of ampulla of Vater | Non-infectious |
| C25.0 | Malignant neoplasm of head of pancreas | Non-infectious |
| C25.1 | Malignant neoplasm of body of pancreas | Non-infectious |
| C25.2 | Malignant neoplasm of tail of pancreas | Non-infectious |
| C25.3 | Malignant neoplasm of pancreatic duct | Non-infectious |
| C25.7 | Malignant neoplasm of other parts of pancreas | Non-infectious |
| C25.8 | Malignant neoplasm of overlapping sites of pancreas | Non-infectious |
| C25.9 | Malignant neoplasm of pancreas, unspecified | Non-infectious |
| C26.9 | Malignant neoplasm of ill-defined sites within the digestive system | Non-infectious |
| C30.0 | Malignant neoplasm of nasal cavity | Non-infectious |
| C31.0 | Malignant neoplasm of maxillary sinus | Non-infectious |
| C32.0 | Malignant neoplasm of glottis | Non-infectious |
| C32.1 | Malignant neoplasm of supraglottis | Non-infectious |
| C32.8 | Malignant neoplasm of overlapping sites of larynx | Non-infectious |
| C32.9 | Malignant neoplasm of larynx, unspecified | Non-infectious |
| C33 | Malignant neoplasm of trachea | Non-infectious |
| C34.01 | Malignant neoplasm of right main bronchus | Non-infectious |
| C34.02 | Malignant neoplasm of left main bronchus | Non-infectious |
| C34.11 | Malignant neoplasm of upper lobe, right bronchus or lung | Non-infectious |
| C34.12 | Malignant neoplasm of upper lobe, left bronchus or lung | Non-infectious |
| C34.2 | Malignant neoplasm of middle lobe, bronchus or lung | Non-infectious |
| C34.31 | Malignant neoplasm of lower lobe, right bronchus or lung | Non-infectious |
| C34.32 | Malignant neoplasm of lower lobe, left bronchus or lung | Non-infectious |
| C34.81 | Malignant neoplasm of overlapping sites of right bronchus and lung | Non-infectious |
| C34.90 | Malignant neoplasm of unspecified part of unspecified bronchus or lung | Non-infectious |
| C34.91 | Malignant neoplasm of unspecified part of right bronchus or lung | Non-infectious |
| C34.92 | Malignant neoplasm of unspecified part of left bronchus or lung | Non-infectious |
| C37 | Malignant neoplasm of thymus | Non-infectious |
| C41.0 | Malignant neoplasm of bones of skull and face | Non-infectious |
| C41.1 | Malignant neoplasm of mandible | Non-infectious |
| C41.2 | Malignant neoplasm of vertebral column | Non-infectious |
| C41.4 | Malignant neoplasm of pelvic bones, sacrum and coccyx | Non-infectious |
| C43.39 | Malignant melanoma of other parts of face | Non-infectious |
| C44.1121 | Basal cell carcinoma of skin of right upper eyelid, including canthus | Non-infectious |
| C44.212 | Basal cell carcinoma of skin of right ear and external auricular canal | Non-infectious |
| C44.222 | Squamous cell carcinoma of skin of right ear and external auricular canal | Non-infectious |
| C44.319 | Basal cell carcinoma of skin of other parts of face | Non-infectious |
| C44.329 | Squamous cell carcinoma of skin of other parts of face | Non-infectious |
| C44.42 | Squamous cell carcinoma of skin of scalp and neck | Non-infectious |
| C44.529 | Squamous cell carcinoma of skin of other part of trunk | Non-infectious |
| C44.629 | Squamous cell carcinoma of skin of left upper limb, including shoulder | Non-infectious |
| C45.0 | Mesothelioma of pleura | Non-infectious |
| C45.1 | Mesothelioma of peritoneum | Non-infectious |
| C48.0 | Malignant neoplasm of retroperitoneum | Non-infectious |
| C48.8 | Malignant neoplasm of overlapping sites of retroperitoneum and peritoneum | Non-infectious |
| C49.0 | Malignant neoplasm of connective and soft tissue of head, face and neck | Non-infectious |
| C49.11 | Malignant neoplasm of connective and soft tissue of right upper limb, including shoulder | Non-infectious |
| C49.21 | Malignant neoplasm of connective and soft tissue of right lower limb, including hip | Non-infectious |
| C49.22 | Malignant neoplasm of connective and soft tissue of left lower limb, including hip | Non-infectious |
| C49.3 | Malignant neoplasm of connective and soft tissue of thorax | Non-infectious |
| C49.5 | Malignant neoplasm of connective and soft tissue of pelvis | Non-infectious |
| C49.6 | Malignant neoplasm of connective and soft tissue of trunk, unspecified | Non-infectious |
| C49.8 | Malignant neoplasm of overlapping sites of connective and soft tissue | Non-infectious |
| C49.9 | Malignant neoplasm of connective and soft tissue, unspecified | Non-infectious |
| C49.A1 | Gastrointestinal stromal tumor of esophagus | Non-infectious |
| C49.A2 | Gastrointestinal stromal tumor of stomach | Non-infectious |
| C49.A3 | Gastrointestinal stromal tumor of small intestine | Non-infectious |
| C49.A5 | Gastrointestinal stromal tumor of rectum | Non-infectious |
| C4A.61 | Merkel cell carcinoma of right upper limb, including shoulder | Non-infectious |
| C50.212 | Malignant neoplasm of upper-inner quadrant of left female breast | Non-infectious |
| C50.312 | Malignant neoplasm of lower-inner quadrant of left female breast | Non-infectious |
| C50.411 | Malignant neoplasm of upper-outer quadrant of right female breast | Non-infectious |
| C50.412 | Malignant neoplasm of upper-outer quadrant of left female breast | Non-infectious |
| C50.811 | Malignant neoplasm of overlapping sites of right female breast | Non-infectious |
| C50.812 | Malignant neoplasm of overlapping sites of left female breast | Non-infectious |
| C50.912 | Malignant neoplasm of unspecified site of left female breast | Non-infectious |
| C51.8 | Malignant neoplasm of overlapping sites of vulva | Non-infectious |
| C51.9 | Malignant neoplasm of vulva, unspecified | Non-infectious |
| C52 | Malignant neoplasm of vagina | Non-infectious |
| C53.0 | Malignant neoplasm of endocervix | Non-infectious |
| C53.1 | Malignant neoplasm of exocervix | Non-infectious |
| C53.9 | Malignant neoplasm of cervix uteri, unspecified | Non-infectious |
| C54.1 | Malignant neoplasm of endometrium | Non-infectious |
| C55 | Malignant neoplasm of uterus, part unspecified | Non-infectious |
| C56.1 | Malignant neoplasm of right ovary | Non-infectious |
| C56.2 | Malignant neoplasm of left ovary | Non-infectious |
| C56.3 | Malignant neoplasm of bilateral ovaries | Non-infectious |
| C56.9 | Malignant neoplasm of unspecified ovary | Non-infectious |
| C57.01 | Malignant neoplasm of right fallopian tube | Non-infectious |
| C57.02 | Malignant neoplasm of left fallopian tube | Non-infectious |
| C57.7 | Malignant neoplasm of other specified female genital organs | Non-infectious |
| C61 | Malignant neoplasm of prostate | Non-infectious |
| C64.1 | Malignant neoplasm of right kidney, except renal pelvis | Non-infectious |
| C64.2 | Malignant neoplasm of left kidney, except renal pelvis | Non-infectious |
| C66.1 | Malignant neoplasm of right ureter | Non-infectious |
| C67.0 | Malignant neoplasm of trigone of bladder | Non-infectious |
| C67.3 | Malignant neoplasm of anterior wall of bladder | Non-infectious |
| C67.4 | Malignant neoplasm of posterior wall of bladder | Non-infectious |
| C67.6 | Malignant neoplasm of ureteric orifice | Non-infectious |
| C67.8 | Malignant neoplasm of overlapping sites of bladder | Non-infectious |
| C67.9 | Malignant neoplasm of bladder, unspecified | Non-infectious |
| C71.2 | Malignant neoplasm of temporal lobe | Non-infectious |
| C72.0 | Malignant neoplasm of spinal cord | Non-infectious |
| C73 | Malignant neoplasm of thyroid gland | Non-infectious |
| C76.2 | Malignant neoplasm of abdomen | Non-infectious |
| C77.0 | Secondary and unspecified malignant neoplasm of lymph nodes of head, face and neck | Non-infectious |
| C77.1 | Secondary and unspecified malignant neoplasm of intrathoracic lymph nodes | Non-infectious |
| C77.2 | Secondary and unspecified malignant neoplasm of intra-abdominal lymph nodes | Non-infectious |
| C77.3 | Secondary and unspecified malignant neoplasm of axilla and upper limb lymph nodes | Non-infectious |
| C77.8 | Secondary and unspecified malignant neoplasm of lymph nodes of multiple regions | Non-infectious |
| C78.00 | Secondary malignant neoplasm of unspecified lung | Non-infectious |
| C78.01 | Secondary malignant neoplasm of right lung | Non-infectious |
| C78.02 | Secondary malignant neoplasm of left lung | Non-infectious |
| C78.1 | Secondary malignant neoplasm of mediastinum | Non-infectious |
| C78.2 | Secondary malignant neoplasm of pleura | Non-infectious |
| C78.4 | Secondary malignant neoplasm of small intestine | Non-infectious |
| C78.6 | Secondary malignant neoplasm of retroperitoneum and peritoneum | Non-infectious |
| C78.7 | Secondary malignant neoplasm of liver and intrahepatic bile duct | Non-infectious |
| C78.89 | Secondary malignant neoplasm of other digestive organs | Non-infectious |
| C79.01 | Secondary malignant neoplasm of right kidney and renal pelvis | Non-infectious |
| C79.11 | Secondary malignant neoplasm of bladder | Non-infectious |
| C79.2 | Secondary malignant neoplasm of skin | Non-infectious |
| C79.31 | Secondary malignant neoplasm of brain | Non-infectious |
| C79.32 | Secondary malignant neoplasm of cerebral meninges | Non-infectious |
| C79.49 | Secondary malignant neoplasm of other parts of nervous system | Non-infectious |
| C79.51 | Secondary malignant neoplasm of bone | Non-infectious |
| C79.89 | Secondary malignant neoplasm of other specified sites | Non-infectious |
| C7A.021 | Malignant carcinoid tumor of the cecum | Non-infectious |
| C7A.090 | Malignant carcinoid tumor of the bronchus and lung | Non-infectious |
| C7A.1 | Malignant poorly differentiated neuroendocrine tumors | Non-infectious |
| C7A.8 | Other malignant neuroendocrine tumors | Non-infectious |
| C7B.1 | Secondary Merkel cell carcinoma | Non-infectious |
| C7B.8 | Other secondary neuroendocrine tumors | Non-infectious |
| C81.10 | Nodular sclerosis Hodgkin lymphoma, unspecified site | Non-infectious |
| C81.11 | Nodular sclerosis Hodgkin lymphoma, lymph nodes of head, face, and neck | Non-infectious |
| C81.12 | Nodular sclerosis Hodgkin lymphoma, intrathoracic lymph nodes | Non-infectious |
| C81.18 | Nodular sclerosis Hodgkin lymphoma, lymph nodes of multiple sites | Non-infectious |
| C81.19 | Nodular sclerosis Hodgkin lymphoma, extranodal and solid organ sites | Non-infectious |
| C81.28 | Mixed cellularity Hodgkin lymphoma, lymph nodes of multiple sites | Non-infectious |
| C81.90 | Hodgkin lymphoma, unspecified, unspecified site | Non-infectious |
| C81.93 | Hodgkin lymphoma, unspecified, intra-abdominal lymph nodes | Non-infectious |
| C81.98 | Hodgkin lymphoma, unspecified, lymph nodes of multiple sites | Non-infectious |
| C81.99 | Hodgkin lymphoma, unspecified, extranodal and solid organ sites | Non-infectious |
| C82.04 | Follicular lymphoma grade i, lymph nodes of axilla and upper limb | Non-infectious |
| C82.15 | Follicular lymphoma grade ii, lymph nodes of inguinal region and lower limb | Non-infectious |
| C82.28 | Follicular lymphoma grade III, unspecified, lymph nodes of multiple sites | Non-infectious |
| C83.10 | Mantle cell lymphoma, unspecified site | Non-infectious |
| C83.13 | Mantle cell lymphoma, intra-abdominal lymph nodes | Non-infectious |
| C83.16 | Mantle cell lymphoma, intrapelvic lymph nodes | Non-infectious |
| C83.18 | Mantle cell lymphoma, lymph nodes of multiple sites | Non-infectious |
| C83.19 | Mantle cell lymphoma, extranodal and solid organ sites | Non-infectious |
| C83.30 | Diffuse large B-cell lymphoma, unspecified site | Non-infectious |
| C83.31 | Diffuse large b-cell lymphoma, lymph nodes of head, face, and neck | Non-infectious |
| C83.32 | Diffuse large b-cell lymphoma, intrathoracic lymph nodes | Non-infectious |
| C83.33 | Diffuse large b-cell lymphoma, intra-abdominal lymph nodes | Non-infectious |
| C83.35 | Diffuse large b-cell lymphoma, lymph nodes of inguinal region and lower limb | Non-infectious |
| C83.36 | Diffuse large b-cell lymphoma, intrapelvic lymph nodes | Non-infectious |
| C83.38 | Diffuse large b-cell lymphoma, lymph nodes of multiple sites | Non-infectious |
| C83.39 | Diffuse large b-cell lymphoma, extranodal and solid organ sites | Non-infectious |
| C83.88 | Other non-follicular lymphoma, lymph nodes of multiple sites | Non-infectious |
| C84.09 | Mycosis fungoides, extranodal and solid organ sites | Non-infectious |
| C84.49 | Peripheral t-cell lymphoma, not elsewhere classified, extranodal and solid organ sites | Non-infectious |
| C84.61 | Anaplastic large cell lymphoma, ALK-positive, lymph nodes of head, face, and neck | Non-infectious |
| C84.98 | Mature T/NK-cell lymphomas, unspecified, lymph nodes of multiple sites | Non-infectious |
| C84.99 | Mature T/NK-cell lymphomas, unspecified, extranodal and solid organ sites | Non-infectious |
| C85.19 | Unspecified b-cell lymphoma, extranodal and solid organ sites | Non-infectious |
| C85.93 | Non-hodgkin lymphoma, unspecified, intra-abdominal lymph nodes | Non-infectious |
| C85.99 | Non-Hodgkin lymphoma, unspecified, extranodal and solid organ sites | Non-infectious |
| C86.1 | Hepatosplenic T-cell lymphoma | Non-infectious |
| C86.2 | Enteropathy-type (intestinal) T-cell lymphoma | Non-infectious |
| C86.4 | Blastic NK-cell lymphoma | Non-infectious |
| C86.5 | Angioimmunoblastic T-cell lymphoma | Non-infectious |
| C88.0 | Waldenstrom macroglobulinemia | Non-infectious |
| C90.00 | Multiple myeloma not having achieved remission | Non-infectious |
| C90.01 | Multiple myeloma in remission | Non-infectious |
| C90.02 | Multiple myeloma in relapse | Non-infectious |
| C91.00 | Acute lymphoblastic leukemia not having achieved remission | Non-infectious |
| C91.01 | Acute lymphoblastic leukemia, in remission | Non-infectious |
| C91.02 | Acute lymphoblastic leukemia, in relapse | Non-infectious |
| C91.10 | Chronic lymphocytic leukemia of B-cell type not having achieved remission | Non-infectious |
| C91.60 | Prolymphocytic leukemia of T-cell type not having achieved remission | Non-infectious |
| C92.00 | Acute myeloblastic leukemia, not having achieved remission | Non-infectious |
| C92.01 | Acute myeloblastic leukemia, in remission | Non-infectious |
| C92.02 | Acute myeloblastic leukemia, in relapse | Non-infectious |
| C92.10 | Chronic myeloid leukemia, BCR/ABL-positive, not having achieved remission | Non-infectious |
| C92.40 | Acute promyelocytic leukemia, not having achieved remission | Non-infectious |
| C92.42 | Acute promyelocytic leukemia, in relapse | Non-infectious |
| C92.50 | Acute myelomonocytic leukemia, not having achieved remission | Non-infectious |
| C93.00 | Acute monoblastic/monocytic leukemia, not having achieved remission | Non-infectious |
| C93.10 | Chronic myelomonocytic leukemia not having achieved remission | Non-infectious |
| C93.11 | Chronic myelomonocytic leukemia, in remission | Non-infectious |
| C94.01 | Acute erythroid leukemia, in remission | Non-infectious |
| C95.00 | Acute leukemia of unspecified cell type not having achieved remission | Non-infectious |
| D05.12 | Intraductal carcinoma in situ of left breast | Non-infectious |
| D05.91 | Unspecified type of carcinoma in situ of right breast | Non-infectious |
| D09.0 | Carcinoma in situ of bladder | Non-infectious |
| D13.2 | Benign neoplasm of duodenum | Non-infectious |
| D13.6 | Benign neoplasm of pancreas | Non-infectious |
| D14.2 | Benign neoplasm of trachea | Non-infectious |
| D16.5 | Benign neoplasm of lower jaw bone | Non-infectious |
| D17.71 | Benign lipomatous neoplasm of kidney | Non-infectious |
| D25.0 | Submucous leiomyoma of uterus | Non-infectious |
| D25.1 | Intramural leiomyoma of uterus | Non-infectious |
| D27.1 | Benign neoplasm of left ovary | Non-infectious |
| D30.01 | Benign neoplasm of right kidney | Non-infectious |
| D32.0 | Benign neoplasm of cerebral meninges | Non-infectious |
| D35.2 | Benign neoplasm of pituitary gland | Non-infectious |
| D36.10 | Benign neoplasm of peripheral nerves and autonomic nervous system, unspecified | Non-infectious |
| D36.14 | Benign neoplasm of peripheral nerves and autonomic nervous system of thorax | Non-infectious |
| D37.8 | Neoplasm of uncertain behavior of other specified digestive organs | Non-infectious |
| D3A.8 | Other benign neuroendocrine tumors | Non-infectious |
| D44.4 | Neoplasm of uncertain behavior of craniopharyngeal duct | Non-infectious |
| D44.6 | Neoplasm of uncertain behavior of carotid body | Non-infectious |
| D44.7 | Neoplasm of uncertain behavior of aortic body and other paraganglia | Non-infectious |
| D46.9 | Myelodysplastic syndrome, unspecified | Non-infectious |
| D46.A | Refractory cytopenia with multilineage dysplasia | Non-infectious |
| D48.1 | Neoplasm of uncertain behavior of connective and other soft tissue | Non-infectious |
| D49.0 | Neoplasm of unspecified behavior of digestive system | Non-infectious |
| D49.2 | Neoplasm of unspecified behavior of bone, soft tissue, and skin | Non-infectious |
| D49.6 | Neoplasm of unspecified behavior of brain | Non-infectious |
| D49.89 | Neoplasm of unspecified behavior of other specified sites | Non-infectious |
| D50.0 | Iron deficiency anemia secondary to blood loss (chronic) | Non-infectious |
| D50.9 | Iron deficiency anemia, unspecified | Non-infectious |
| D57.00 | Hb-SS disease with crisis, unspecified | Non-infectious |
| D57.01 | Hb-SS disease with acute chest syndrome | Non-infectious |
| D57.218 | Sickle-cell/hb-c disease with crisis with other specified complication | Non-infectious |
| D57.40 | Sickle-cell thalassemia without crisis | Non-infectious |
| D57.459 | Sickle-cell thalassemia beta plus with crisis, unspecified | Non-infectious |
| D59.13 | Mixed type autoimmune hemolytic anemia | Non-infectious |
| D61.01 | Constitutional (pure) red blood cell aplasia | Non-infectious |
| D61.1 | Drug-induced aplastic anemia | Non-infectious |
| D61.810 | Antineoplastic chemotherapy induced pancytopenia | Non-infectious |
| D61.818 | Other pancytopenia | Non-infectious |
| D61.9 | Aplastic anemia, unspecified | Non-infectious |
| D62 | Acute posthemorrhagic anemia | Non-infectious |
| D64.81 | Anemia due to antineoplastic chemotherapy | Non-infectious |
| D64.9 | Anemia, unspecified | Non-infectious |
| D68.32 | Hemorrhagic disorder due to extrinsic circulating anticoagulants | Non-infectious |
| D69.3 | Immune thrombocytopenic purpura | Non-infectious |
| D69.59 | Other secondary thrombocytopenia | Non-infectious |
| D69.6 | Thrombocytopenia, unspecified | Non-infectious |
| D70.1 | Agranulocytosis secondary to cancer chemotherapy | Non-infectious |
| D70.2 | Other drug-induced agranulocytosis | Non-infectious |
| D70.3 | Neutropenia due to infection | Other |
| D70.4 | Cyclic neutropenia | Non-infectious |
| D70.8 | Other neutropenia | Non-infectious |
| D70.9 | Neutropenia, unspecified | Non-infectious |
| D72.12 | Drug rash with eosinophilia and systemic symptoms syndrome | Non-infectious |
| D72.829 | Elevated white blood cell count, unspecified | Non-infectious |
| D73.3 | Abscess of spleen | Abdomen/pelvis |
| D73.5 | Infarction of spleen | Non-infectious |
| D75.81 | Myelofibrosis | Non-infectious |
| D76.1 | Hemophagocytic lymphohistiocytosis | Non-infectious |
| D76.3 | Other histiocytosis syndromes | Non-infectious |
| D83.9 | Common variable immunodeficiency, unspecified | Non-infectious |
| D86.0 | Sarcoidosis of lung | Non-infectious |
| D89.1 | Cryoglobulinemia | Non-infectious |
| E03.8 | Other specified hypothyroidism | Non-infectious |
| E05.20 | Thyrotoxicosis with toxic multinodular goiter without thyrotoxic crisis or storm | Non-infectious |
| E05.90 | Thyrotoxicosis, unspecified without thyrotoxic crisis or storm | Non-infectious |
| E10.10 | Type 1 diabetes mellitus with ketoacidosis without coma | Non-infectious |
| E10.43 | Type 1 diabetes mellitus with diabetic autonomic (poly)neuropathy | Non-infectious |
| E10.51 | Type 1 diabetes mellitus with diabetic peripheral angiopathy without gangrene | Non-infectious |
| E10.621 | Type 1 diabetes mellitus with foot ulcer | SSTI |
| E10.649 | Type 1 diabetes mellitus with hypoglycemia without coma | Non-infectious |
| E10.65 | Type 1 diabetes mellitus with hyperglycemia | Non-infectious |
| E10.69 | Type 1 diabetes mellitus with other specified complication | Non-infectious |
| E11.00 | Type 2 diabetes mellitus with hyperosmolarity without nonketotic hyperglycemic-hyperosmolar coma (NKHHC) | Non-infectious |
| E11.10 | Type 2 diabetes mellitus with ketoacidosis without coma | Non-infectious |
| E11.22 | Type 2 diabetes mellitus with diabetic chronic kidney disease | Non-infectious |
| E11.3593 | Type 2 diabetes mellitus with proliferative diabetic retinopathy without macular edema, bilateral | Non-infectious |
| E11.39 | Type 2 diabetes mellitus with other diabetic ophthalmic complication | Non-infectious |
| E11.40 | Type 2 diabetes mellitus with diabetic neuropathy, unspecified | Non-infectious |
| E11.43 | Type 2 diabetes mellitus with diabetic autonomic (poly)neuropathy | Non-infectious |
| E11.51 | Type 2 diabetes mellitus with diabetic peripheral angiopathy without gangrene | Non-infectious |
| E11.52 | Type 2 diabetes mellitus with diabetic peripheral angiopathy with gangrene | SSTI |
| E11.621 | Type 2 diabetes mellitus with foot ulcer | Non-infectious |
| E11.622 | Type 2 diabetes mellitus with other skin ulcer | Non-infectious |
| E11.628 | Type 2 diabetes mellitus with other skin complications | Non-infectious |
| E11.641 | Type 2 diabetes mellitus with hypoglycemia with coma | Non-infectious |
| E11.649 | Type 2 diabetes mellitus with hypoglycemia without coma | Non-infectious |
| E11.65 | Type 2 diabetes mellitus with hyperglycemia | Non-infectious |
| E11.69 | Type 2 diabetes mellitus with other specified complication | Non-infectious |
| E16.2 | Hypoglycemia, unspecified | Non-infectious |
| E22.2 | Syndrome of inappropriate secretion of antidiuretic hormone | Non-infectious |
| E24.9 | Cushing's syndrome, unspecified | Non-infectious |
| E27.2 | Addisonian crisis | Non-infectious |
| E27.3 | Drug-induced adrenocortical insufficiency | Non-infectious |
| E27.40 | Unspecified adrenocortical insufficiency | Non-infectious |
| E27.49 | Other adrenocortical insufficiency | Non-infectious |
| E43 | Unspecified severe protein-calorie malnutrition | Non-infectious |
| E44.0 | Moderate protein-calorie malnutrition | Non-infectious |
| E66.01 | Morbid (severe) obesity due to excess calories | Non-infectious |
| E83.39 | Other disorders of phosphorus metabolism | Non-infectious |
| E83.52 | Hypercalcemia | Non-infectious |
| E84.0 | Cystic fibrosis with pulmonary manifestations | Non-infectious |
| E84.8 | Cystic fibrosis with other manifestations | Non-infectious |
| E85.4 | Organ-limited amyloidosis | Non-infectious |
| E85.81 | Light chain (AL) amyloidosis | Non-infectious |
| E85.9 | Amyloidosis, unspecified | Non-infectious |
| E86.0 | Dehydration | Non-infectious |
| E86.1 | Hypovolemia | Non-infectious |
| E86.9 | Volume depletion, unspecified | Non-infectious |
| E87.1 | Hypo-osmolality and hyponatremia | Non-infectious |
| E87.2 | Acidosis | Non-infectious |
| E87.20 | Acidosis, unspecified | Non-infectious |
| E87.29 | Other acidosis | Non-infectious |
| E87.5 | Hyperkalemia | Non-infectious |
| E87.6 | Hypokalemia | Non-infectious |
| E87.70 | Fluid overload, unspecified | Non-infectious |
| E87.71 | Transfusion associated circulatory overload | Non-infectious |
| E87.79 | Other fluid overload | Non-infectious |
| E88.09 | Other disorders of plasma-protein metabolism, not elsewhere classified | Non-infectious |
| E88.3 | Tumor lysis syndrome | Non-infectious |
| F01.50 | Vascular dementia, unspecified severity, without behavioral disturbance, psychotic disturbance, mood disturbance, and anxiety | Non-infectious |
| F01.51 | Vascular dementia, unspecified severity, with behavioral disturbance | Non-infectious |
| F03.90 | Unspecified dementia, unspecified severity, without behavioral disturbance, psychotic disturbance, mood disturbance, and anxiety | Non-infectious |
| F03.92 | Unspecified dementia, unspecified severity, with psychotic disturbance | Non-infectious |
| F03.B0 | Unspecified dementia, moderate, without behavioral disturbance, psychotic disturbance, mood disturbance, and anxiety | Non-infectious |
| F10.130 | Alcohol abuse with withdrawal, uncomplicated | Non-infectious |
| F10.131 | Alcohol abuse with withdrawal delirium | Non-infectious |
| F10.139 | Alcohol abuse with withdrawal, unspecified | Non-infectious |
| F10.229 | Alcohol dependence with intoxication, unspecified | Non-infectious |
| F10.230 | Alcohol dependence with withdrawal, uncomplicated | Non-infectious |
| F10.231 | Alcohol dependence with withdrawal delirium | Non-infectious |
| F10.232 | Alcohol dependence with withdrawal with perceptual disturbance | Non-infectious |
| F10.239 | Alcohol dependence with withdrawal, unspecified | Non-infectious |
| F10.288 | Alcohol dependence with other alcohol-induced disorder | Non-infectious |
| F11.23 | Opioid dependence with withdrawal | Non-infectious |
| F15.10 | Other stimulant abuse, uncomplicated | Non-infectious |
| F15.129 | Other stimulant abuse with intoxication, unspecified | Non-infectious |
| F15.90 | Other stimulant use, unspecified, uncomplicated | Non-infectious |
| F15.929 | Other stimulant use, unspecified with intoxication, unspecified | Non-infectious |
| F20.0 | Paranoid schizophrenia | Non-infectious |
| F22 | Delusional disorders | Non-infectious |
| F29 | Unspecified psychosis not due to a substance or known physiological condition | Non-infectious |
| F32.3 | Major depressive disorder, single episode, severe with psychotic features | Non-infectious |
| F41.9 | Anxiety disorder, unspecified | Non-infectious |
| F44.4 | Conversion disorder with motor symptom or deficit | Non-infectious |
| F44.5 | Conversion disorder with seizures or convulsions | Non-infectious |
| F45.41 | Pain disorder exclusively related to psychological factors | Non-infectious |
| G04.81 | Other encephalitis and encephalomyelitis | CNS/meningitis |
| G04.90 | Encephalitis and encephalomyelitis, unspecified | CNS/meningitis |
| G06.0 | Intracranial abscess and granuloma | CNS/meningitis |
| G06.1 | Intraspinal abscess and granuloma | CNS/meningitis |
| G06.2 | Extradural and subdural abscess, unspecified | CNS/meningitis |
| G12.21 | Amyotrophic lateral sclerosis | Non-infectious |
| G20 | Parkinson's disease | Non-infectious |
| G20.A1 | Parkinson's disease without dyskinesia, without mention of fluctuations | Non-infectious |
| G25.0 | Essential tremor | Non-infectious |
| G25.3 | Myoclonus | Non-infectious |
| G30.1 | Alzheimer's disease with late onset | Non-infectious |
| G30.9 | Alzheimer's disease, unspecified | Non-infectious |
| G31.89 | Other specified degenerative diseases of nervous system | Non-infectious |
| G35 | Multiple sclerosis | Non-infectious |
| G36.0 | Neuromyelitis optica (devic) | Non-infectious |
| G37.3 | Acute transverse myelitis in demyelinating disease of central nervous system | Non-infectious |
| G40.009 | Localization-related (focal) (partial) idiopathic epilepsy and epileptic syndromes with seizures of localized onset, not intractable, without status epilepticus | Non-infectious |
| G40.011 | Localization-related (focal) (partial) idiopathic epilepsy and epileptic syndromes with seizures of localized onset, intractable, with status epilepticus | Non-infectious |
| G40.101 | Localization-related (focal) (partial) symptomatic epilepsy and epileptic syndromes with simple partial seizures, not intractable, with status epilepticus | Non-infectious |
| G40.109 | Localization-related (focal) (partial) symptomatic epilepsy and epileptic syndromes with simple partial seizures, not intractable, without status epilepticus | Non-infectious |
| G40.119 | Localization-related (focal) (partial) symptomatic epilepsy and epileptic syndromes with simple partial seizures, intractable, without status epilepticus | Non-infectious |
| G40.201 | Localization-related (focal) (partial) symptomatic epilepsy and epileptic syndromes with complex partial seizures, not intractable, with status epilepticus | Non-infectious |
| G40.401 | Other generalized epilepsy and epileptic syndromes, not intractable, with status epilepticus | Non-infectious |
| G40.409 | Other generalized epilepsy and epileptic syndromes, not intractable, without status epilepticus | Non-infectious |
| G40.419 | Other generalized epilepsy and epileptic syndromes, intractable, without status epilepticus | Non-infectious |
| G40.509 | Epileptic seizures related to external causes, not intractable, without status epilepticus | Non-infectious |
| G40.801 | Other epilepsy, not intractable, with status epilepticus | Non-infectious |
| G40.89 | Other seizures | Non-infectious |
| G40.901 | Epilepsy, unspecified, not intractable, with status epilepticus | Non-infectious |
| G40.909 | Epilepsy, unspecified, not intractable, without status epilepticus | Non-infectious |
| G40.919 | Epilepsy, unspecified, intractable, without status epilepticus | Non-infectious |
| G43.909 | Migraine, unspecified, not intractable, without status migrainosus | Non-infectious |
| G44.89 | Other headache syndrome | Non-infectious |
| G45.9 | Transient cerebral ischemic attack, unspecified | Non-infectious |
| G47.33 | Obstructive sleep apnea (adult) (pediatric) | Non-infectious |
| G50.0 | Trigeminal neuralgia | Non-infectious |
| G50.1 | Atypical facial pain | Non-infectious |
| G51.0 | Bell's palsy | Non-infectious |
| G57.02 | Lesion of sciatic nerve, left lower limb | Non-infectious |
| G60.3 | Idiopathic progressive neuropathy | Non-infectious |
| G61.0 | Guillain-Barre syndrome | Non-infectious |
| G61.81 | Chronic inflammatory demyelinating polyneuritis | Non-infectious |
| G62.89 | Other specified polyneuropathies | Non-infectious |
| G62.9 | Polyneuropathy, unspecified | Non-infectious |
| G70.00 | Myasthenia gravis without (acute) exacerbation | Non-infectious |
| G70.01 | Myasthenia gravis with (acute) exacerbation | Non-infectious |
| G72.0 | Drug-induced myopathy | Non-infectious |
| G83.84 | Todd's paralysis (postepileptic) | Non-infectious |
| G89.18 | Other acute postprocedural pain | Non-infectious |
| G89.29 | Other chronic pain | Non-infectious |
| G89.3 | Neoplasm related pain (acute) (chronic) | Non-infectious |
| G90.1 | Familial dysautonomia (riley-day) | Non-infectious |
| G90.3 | Multi-system degeneration of the autonomic nervous system | Non-infectious |
| G90.523 | Complex regional pain syndrome i of lower limb, bilateral | Non-infectious |
| G91.0 | Communicating hydrocephalus | Non-infectious |
| G91.1 | Obstructive hydrocephalus | Non-infectious |
| G91.2 | (Idiopathic) normal pressure hydrocephalus | Non-infectious |
| G91.3 | Post-traumatic hydrocephalus, unspecified | Non-infectious |
| G91.9 | Hydrocephalus, unspecified | Non-infectious |
| G92 | Toxic encephalopathy | Non-infectious |
| G92.8 | Other toxic encephalopathy | Non-infectious |
| G93.0 | Cerebral cysts | Non-infectious |
| G93.40 | Encephalopathy, unspecified | Non-infectious |
| G93.41 | Metabolic encephalopathy | Non-infectious |
| G93.49 | Other encephalopathy | Non-infectious |
| G93.5 | Compression of brain | Non-infectious |
| G93.6 | Cerebral edema | Non-infectious |
| G93.89 | Other specified disorders of brain | Non-infectious |
| G93.9 | Disorder of brain, unspecified | Non-infectious |
| G95.20 | Unspecified cord compression | Non-infectious |
| G95.29 | Other cord compression | Non-infectious |
| G95.89 | Other specified diseases of spinal cord | Non-infectious |
| G95.9 | Disease of spinal cord, unspecified | Non-infectious |
| G96.00 | Cerebrospinal fluid leak, unspecified | Non-infectious |
| G96.198 | Other disorders of meninges, not elsewhere classified | Non-infectious |
| G97.1 | Other reaction to spinal and lumbar puncture | Non-infectious |
| G97.82 | Other postprocedural complications and disorders of nervous system | Non-infectious |
| H00.11 | Chalazion right upper eyelid | Non-infectious |
| H04.322 | Acute dacryocystitis of left lacrimal passage | Other |
| H05.00 | Unspecified acute inflammation of orbit | SSTI |
| H05.011 | Cellulitis of right orbit | SSTI |
| H05.012 | Cellulitis of left orbit | SSTI |
| H05.021 | Osteomyelitis of right orbit | bone/joint infections |
| H11.32 | Conjunctival hemorrhage, left eye | Non-infectious |
| H16.001 | Unspecified corneal ulcer, right eye | Non-infectious |
| H16.071 | Perforated corneal ulcer, right eye | Non-infectious |
| H40.052 | Ocular hypertension, left eye | Non-infectious |
| H44.002 | Unspecified purulent endophthalmitis, left eye | Other |
| H44.011 | Panophthalmitis (acute), right eye | Other |
| H44.111 | Panuveitis, right eye | Other |
| H46.9 | Unspecified optic neuritis | Non-infectious |
| H47.013 | Ischemic optic neuropathy, bilateral | Non-infectious |
| H47.10 | Unspecified papilledema | Non-infectious |
| H60.11 | Cellulitis of right external ear | SSTI |
| H60.21 | Malignant otitis externa, right ear | Other |
| H60.391 | Other infective otitis externa, right ear | Other |
| H60.501 | Unspecified acute noninfective otitis externa, right ear | Other |
| H60.8X1 | Other otitis externa, right ear | Other |
| H60.92 | Unspecified otitis externa, left ear | Other |
| H61.002 | Unspecified perichondritis of left external ear | Other |
| H61.032 | Chondritis of left external ear | Other |
| H66.92 | Otitis media, unspecified, left ear | Other |
| H70.002 | Acute mastoiditis without complications, left ear | bone/joint infections |
| H70.092 | Acute mastoiditis with other complications, left ear | bone/joint infections |
| H70.892 | Other mastoiditis and related conditions, left ear | bone/joint infections |
| H70.92 | Unspecified mastoiditis, left ear | bone/joint infections |
| H74.8X2 | Other specified disorders of left middle ear and mastoid | Non-infectious |
| H81.11 | Benign paroxysmal vertigo, right ear | Non-infectious |
| H93.3X1 | Disorders of right acoustic nerve | Non-infectious |
| H95.01 | Recurrent cholesteatoma of postmastoidectomy cavity, right ear | Non-infectious |
| I05.0 | Rheumatic mitral stenosis | Non-infectious |
| I05.2 | Rheumatic mitral stenosis with insufficiency | Non-infectious |
| I07.1 | Rheumatic tricuspid insufficiency | Non-infectious |
| I08.0 | Rheumatic disorders of both mitral and aortic valves | Non-infectious |
| I08.1 | Rheumatic disorders of both mitral and tricuspid valves | Non-infectious |
| I08.2 | Rheumatic disorders of both aortic and tricuspid valves | Non-infectious |
| I08.3 | Combined rheumatic disorders of mitral, aortic and tricuspid valves | Non-infectious |
| I08.8 | Other rheumatic multiple valve diseases | Non-infectious |
| I11.0 | Hypertensive heart disease with heart failure | Non-infectious |
| I12.0 | Hypertensive chronic kidney disease with stage 5 chronic kidney disease or end stage renal disease | Non-infectious |
| I13.0 | Hypertensive heart and chronic kidney disease with heart failure and stage 1 through stage 4 chronic kidney disease, or unspecified chronic kidney disease | Non-infectious |
| I13.2 | Hypertensive heart and chronic kidney disease with heart failure and with stage 5 chronic kidney disease, or end stage renal disease | Non-infectious |
| I16.1 | Hypertensive emergency | Non-infectious |
| I20.9 | Angina pectoris, unspecified | Non-infectious |
| I21.01 | ST elevation (STEMI) myocardial infarction involving left main coronary artery | Non-infectious |
| I21.11 | ST elevation (STEMI) myocardial infarction involving right coronary artery | Non-infectious |
| I21.19 | ST elevation (STEMI) myocardial infarction involving other coronary artery of inferior wall | Non-infectious |
| I21.3 | ST elevation (STEMI) myocardial infarction of unspecified site | Non-infectious |
| I21.4 | Non-ST elevation (NSTEMI) myocardial infarction | Non-infectious |
| I21.A1 | Myocardial infarction type 2 | Non-infectious |
| I24.8 | Other forms of acute ischemic heart disease | Non-infectious |
| I25.10 | Atherosclerotic heart disease of native coronary artery without angina pectoris | Non-infectious |
| I25.110 | Atherosclerotic heart disease of native coronary artery with unstable angina pectoris | Non-infectious |
| I25.118 | Atherosclerotic heart disease of native coronary artery with other forms of angina pectoris | Non-infectious |
| I25.119 | Atherosclerotic heart disease of native coronary artery with unspecified angina pectoris | Non-infectious |
| I25.700 | Atherosclerosis of coronary artery bypass graft(s), unspecified, with unstable angina pectoris | Non-infectious |
| I26.02 | Saddle embolus of pulmonary artery with acute cor pulmonale | Non-infectious |
| I26.09 | Other pulmonary embolism with acute cor pulmonale | Non-infectious |
| I26.92 | Saddle embolus of pulmonary artery without acute cor pulmonale | Non-infectious |
| I26.93 | Single subsegmental pulmonary embolism without acute cor pulmonale | Non-infectious |
| I26.94 | Multiple subsegmental pulmonary emboli without acute cor pulmonale | Non-infectious |
| I26.99 | Other pulmonary embolism without acute cor pulmonale | Non-infectious |
| I27.21 | Secondary pulmonary arterial hypertension | Non-infectious |
| I27.23 | Pulmonary hypertension due to lung diseases and hypoxia | Non-infectious |
| I27.24 | Chronic thromboembolic pulmonary hypertension | Non-infectious |
| I27.83 | Eisenmenger's syndrome | Non-infectious |
| I28.1 | Aneurysm of pulmonary artery | Non-infectious |
| I30.9 | Acute pericarditis, unspecified | Other |
| I31.1 | Chronic constrictive pericarditis | Non-infectious |
| I31.3 | Pericardial effusion (noninflammatory) | Non-infectious |
| I31.39 | Other pericardial effusion (noninflammatory) | Non-infectious |
| I31.9 | Disease of pericardium, unspecified | Non-infectious |
| I33.0 | Acute and subacute infective endocarditis | Endocarditis |
| I34.0 | Nonrheumatic mitral (valve) insufficiency | Non-infectious |
| I34.1 | Nonrheumatic mitral (valve) prolapse | Non-infectious |
| I34.2 | Nonrheumatic mitral (valve) stenosis | Non-infectious |
| I35.0 | Nonrheumatic aortic (valve) stenosis | Non-infectious |
| I35.1 | Nonrheumatic aortic (valve) insufficiency | Non-infectious |
| I35.2 | Nonrheumatic aortic (valve) stenosis with insufficiency | Non-infectious |
| I36.1 | Nonrheumatic tricuspid (valve) insufficiency | Non-infectious |
| I37.1 | Nonrheumatic pulmonary valve insufficiency | Non-infectious |
| I38 | Endocarditis, valve unspecified | Endocarditis |
| I40.0 | Infective myocarditis | Other |
| I40.8 | Other acute myocarditis | Non-infectious |
| I40.9 | Acute myocarditis, unspecified | Non-infectious |
| I42.0 | Dilated cardiomyopathy | Non-infectious |
| I42.1 | Obstructive hypertrophic cardiomyopathy | Non-infectious |
| I42.2 | Other hypertrophic cardiomyopathy | Non-infectious |
| I42.8 | Other cardiomyopathies | Non-infectious |
| I44.1 | Atrioventricular block, second degree | Non-infectious |
| I44.2 | Atrioventricular block, complete | Non-infectious |
| I47.1 | Supraventricular tachycardia | Non-infectious |
| I47.11 | Inappropriate sinus tachycardia, so stated | Non-infectious |
| I47.2 | Ventricular tachycardia | Non-infectious |
| I47.20 | Ventricular tachycardia, unspecified | Non-infectious |
| I48.0 | Paroxysmal atrial fibrillation | Non-infectious |
| I48.11 | Longstanding persistent atrial fibrillation | Non-infectious |
| I48.19 | Other persistent atrial fibrillation | Non-infectious |
| I48.20 | Chronic atrial fibrillation, unspecified | Non-infectious |
| I48.3 | Typical atrial flutter | Non-infectious |
| I48.4 | Atypical atrial flutter | Non-infectious |
| I48.91 | Unspecified atrial fibrillation | Non-infectious |
| I48.92 | Unspecified atrial flutter | Non-infectious |
| I49.01 | Ventricular fibrillation | Non-infectious |
| I49.5 | Sick sinus syndrome | Non-infectious |
| I50.21 | Acute systolic (congestive) heart failure | Non-infectious |
| I50.23 | Acute on chronic systolic (congestive) heart failure | Non-infectious |
| I50.33 | Acute on chronic diastolic (congestive) heart failure | Non-infectious |
| I50.43 | Acute on chronic combined systolic (congestive) and diastolic (congestive) heart failure | Non-infectious |
| I50.813 | Acute on chronic right heart failure | Non-infectious |
| I50.84 | End stage heart failure | Non-infectious |
| I51.3 | Intracardiac thrombosis, not elsewhere classified | Non-infectious |
| I51.81 | Takotsubo syndrome | Non-infectious |
| I60.01 | Nontraumatic subarachnoid hemorrhage from right carotid siphon and bifurcation | Non-infectious |
| I60.02 | Nontraumatic subarachnoid hemorrhage from left carotid siphon and bifurcation | Non-infectious |
| I60.32 | Nontraumatic subarachnoid hemorrhage from left posterior communicating artery | Non-infectious |
| I60.52 | Nontraumatic subarachnoid hemorrhage from left vertebral artery | Non-infectious |
| I60.6 | Nontraumatic subarachnoid hemorrhage from other intracranial arteries | Non-infectious |
| I61.0 | Nontraumatic intracerebral hemorrhage in hemisphere, subcortical | Non-infectious |
| I61.1 | Nontraumatic intracerebral hemorrhage in hemisphere, cortical | Non-infectious |
| I61.3 | Nontraumatic intracerebral hemorrhage in brain stem | Non-infectious |
| I61.4 | Nontraumatic intracerebral hemorrhage in cerebellum | Non-infectious |
| I61.5 | Nontraumatic intracerebral hemorrhage, intraventricular | Non-infectious |
| I62.01 | Nontraumatic acute subdural hemorrhage | Non-infectious |
| I63.132 | Cerebral infarction due to embolism of left carotid artery | Non-infectious |
| I63.29 | Cerebral infarction due to unspecified occlusion or stenosis of other precerebral arteries | Non-infectious |
| I63.311 | Cerebral infarction due to thrombosis of right middle cerebral artery | Non-infectious |
| I63.40 | Cerebral infarction due to embolism of unspecified cerebral artery | Non-infectious |
| I63.411 | Cerebral infarction due to embolism of right middle cerebral artery | Non-infectious |
| I63.412 | Cerebral infarction due to embolism of left middle cerebral artery | Non-infectious |
| I63.432 | Cerebral infarction due to embolism of left posterior cerebral artery | Non-infectious |
| I63.449 | Cerebral infarction due to embolism of unspecified cerebellar artery | Non-infectious |
| I63.49 | Cerebral infarction due to embolism of other cerebral artery | Non-infectious |
| I63.511 | Cerebral infarction due to unspecified occlusion or stenosis of right middle cerebral artery | Non-infectious |
| I63.512 | Cerebral infarction due to unspecified occlusion or stenosis of left middle cerebral artery | Non-infectious |
| I63.523 | Cerebral infarction due to unspecified occlusion or stenosis of bilateral anterior cerebral arteries | Non-infectious |
| I63.541 | Cerebral infarction due to unspecified occlusion or stenosis of right cerebellar artery | Non-infectious |
| I63.549 | Cerebral infarction due to unspecified occlusion or stenosis of unspecified cerebellar artery | Non-infectious |
| I63.81 | Other cerebral infarction due to occlusion or stenosis of small artery | Non-infectious |
| I63.89 | Other cerebral infarction | Non-infectious |
| I63.9 | Cerebral infarction, unspecified | Non-infectious |
| I65.22 | Occlusion and stenosis of left carotid artery | Non-infectious |
| I67.1 | Cerebral aneurysm, nonruptured | Non-infectious |
| I67.4 | Hypertensive encephalopathy | Non-infectious |
| I67.5 | Moyamoya disease | Non-infectious |
| I67.6 | Nonpyogenic thrombosis of intracranial venous system | Non-infectious |
| I67.83 | Posterior reversible encephalopathy syndrome | Non-infectious |
| I67.89 | Other cerebrovascular disease | Non-infectious |
| I69.320 | Aphasia following cerebral infarction | Non-infectious |
| I69.354 | Hemiplegia and hemiparesis following cerebral infarction affecting left non-dominant side | Non-infectious |
| I69.918 | Other symptoms and signs involving cognitive functions following unspecified cerebrovascular disease | Non-infectious |
| I70.222 | Atherosclerosis of native arteries of extremities with rest pain, left leg | Non-infectious |
| I70.223 | Atherosclerosis of native arteries of extremities with rest pain, bilateral legs | Non-infectious |
| I70.232 | Atherosclerosis of native arteries of right leg with ulceration of calf | Non-infectious |
| I70.245 | Atherosclerosis of native arteries of left leg with ulceration of other part of foot | Non-infectious |
| I70.248 | Atherosclerosis of native arteries of left leg with ulceration of other part of lower leg | Non-infectious |
| I70.261 | Atherosclerosis of native arteries of extremities with gangrene, right leg | SSTI |
| I70.262 | Atherosclerosis of native arteries of extremities with gangrene, left leg | SSTI |
| I70.268 | Atherosclerosis of native arteries of extremities with gangrene, other extremity | SSTI |
| I71.00 | Dissection of unspecified site of aorta | Non-infectious |
| I71.01 | Dissection of thoracic aorta | Non-infectious |
| I71.010 | Dissection of ascending aorta | Non-infectious |
| I71.011 | Dissection of aortic arch | Non-infectious |
| I71.012 | Dissection of descending thoracic aorta | Non-infectious |
| I71.019 | Dissection of thoracic aorta, unspecified | Non-infectious |
| I71.03 | Dissection of thoracoabdominal aorta | Non-infectious |
| I71.1 | Thoracic aortic aneurysm, ruptured | Non-infectious |
| I71.13 | Aneurysm of the descending thoracic aorta, ruptured | Non-infectious |
| I71.2 | Thoracic aortic aneurysm, without rupture | Non-infectious |
| I71.21 | Aneurysm of the ascending aorta, without rupture | Non-infectious |
| I71.22 | Aneurysm of the aortic arch, without rupture | Non-infectious |
| I71.23 | Aneurysm of the descending thoracic aorta, without rupture | Non-infectious |
| I71.3 | Abdominal aortic aneurysm, ruptured | Non-infectious |
| I71.4 | Abdominal aortic aneurysm, without rupture | Non-infectious |
| I71.43 | Infrarenal abdominal aortic aneurysm, without rupture | Non-infectious |
| I71.5 | Thoracoabdominal aortic aneurysm, ruptured | Non-infectious |
| I71.6 | Thoracoabdominal aortic aneurysm, without rupture | Non-infectious |
| I71.60 | Thoracoabdominal aortic aneurysm, without rupture, unspecified | Non-infectious |
| I72.0 | Aneurysm of carotid artery | Non-infectious |
| I72.3 | Aneurysm of iliac artery | Non-infectious |
| I73.1 | Thromboangiitis obliterans (Buerger's disease) | Non-infectious |
| I74.3 | Embolism and thrombosis of arteries of the lower extremities | Non-infectious |
| I75.023 | Atheroembolism of bilateral lower extremities | Non-infectious |
| I77.0 | Arteriovenous fistula, acquired | Non-infectious |
| I77.71 | Dissection of carotid artery | Non-infectious |
| I77.810 | Thoracic aortic ectasia | Non-infectious |
| I77.82 | Antineutrophilic cytoplasmic antibody (ANCA) vasculitis | Non-infectious |
| I78.0 | Hereditary hemorrhagic telangiectasia | Non-infectious |
| I80.8 | Phlebitis and thrombophlebitis of other sites | Non-infectious |
| I81 | Portal vein thrombosis | Non-infectious |
| I82.220 | Acute embolism and thrombosis of inferior vena cava | Non-infectious |
| I82.221 | Chronic embolism and thrombosis of inferior vena cava | Non-infectious |
| I82.3 | Embolism and thrombosis of renal vein | Non-infectious |
| I82.411 | Acute embolism and thrombosis of right femoral vein | Non-infectious |
| I82.412 | Acute embolism and thrombosis of left femoral vein | Non-infectious |
| I82.413 | Acute embolism and thrombosis of femoral vein, bilateral | Non-infectious |
| I82.422 | Acute embolism and thrombosis of left iliac vein | Non-infectious |
| I82.431 | Acute embolism and thrombosis of right popliteal vein | Non-infectious |
| I82.432 | Acute embolism and thrombosis of left popliteal vein | Non-infectious |
| I82.441 | Acute embolism and thrombosis of right tibial vein | Non-infectious |
| I82.442 | Acute embolism and thrombosis of left tibial vein | Non-infectious |
| I82.463 | Acute embolism and thrombosis of calf muscular vein, bilateral | Non-infectious |
| I82.4Y2 | Acute embolism and thrombosis of unspecified deep veins of left proximal lower extremity | Non-infectious |
| I82.B12 | Acute embolism and thrombosis of left subclavian vein | Non-infectious |
| I83.012 | Varicose veins of right lower extremity with ulcer of calf | Non-infectious |
| I83.015 | Varicose veins of right lower extremity with ulcer other part of foot | Non-infectious |
| I83.209 | Varicose veins of unspecified lower extremity with both ulcer of unspecified site and inflammation | Non-infectious |
| I83.223 | Varicose veins of left lower extremity with both ulcer of ankle and inflammation | Non-infectious |
| I85.01 | Esophageal varices with bleeding | Non-infectious |
| I87.1 | Compression of vein | Non-infectious |
| I87.2 | Venous insufficiency (chronic) (peripheral) | Non-infectious |
| I87.333 | Chronic venous hypertension (idiopathic) with ulcer and inflammation of bilateral lower extremity | Non-infectious |
| I89.0 | Lymphedema, not elsewhere classified | Non-infectious |
| I89.8 | Other specified noninfective disorders of lymphatic vessels and lymph nodes | Non-infectious |
| I95.1 | Orthostatic hypotension | Non-infectious |
| I95.2 | Hypotension due to drugs | Non-infectious |
| I95.3 | Hypotension of hemodialysis | Non-infectious |
| I95.89 | Other hypotension | Non-infectious |
| I95.9 | Hypotension, unspecified | Non-infectious |
| I96 | Gangrene, not elsewhere classified | bone/joint infections |
| I97.89 | Other postprocedural complications and disorders of the circulatory system, not elsewhere classified | Non-infectious |
| J00 | Acute nasopharyngitis (common cold) | Non-infectious |
| J01.00 | Acute maxillary sinusitis, unspecified | Other |
| J01.30 | Acute sphenoidal sinusitis, unspecified | Other |
| J01.40 | Acute pansinusitis, unspecified | Other |
| J01.80 | Other acute sinusitis | Other |
| J01.81 | Other acute recurrent sinusitis | Other |
| J01.91 | Acute recurrent sinusitis, unspecified | Other |
| J02.0 | Streptococcal pharyngitis | Other |
| J02.9 | Acute pharyngitis, unspecified | Other |
| J03.80 | Acute tonsillitis due to other specified organisms | Other |
| J03.90 | Acute tonsillitis, unspecified | Other |
| J03.91 | Acute recurrent tonsillitis, unspecified | Other |
| J04.30 | Supraglottitis, unspecified, without obstruction | Other |
| J05.11 | Acute epiglottitis with obstruction | Other |
| J06.9 | Acute upper respiratory infection, unspecified | Non-infectious |
| J10.00 | Influenza due to other identified influenza virus with unspecified type of pneumonia | Non-infectious |
| J10.01 | Influenza due to other identified influenza virus with the same other identified influenza virus pneumonia | Non-infectious |
| J10.08 | Influenza due to other identified influenza virus with other specified pneumonia | Non-infectious |
| J10.1 | Influenza due to other identified influenza virus with other respiratory manifestations | Non-infectious |
| J11.00 | Influenza due to unidentified influenza virus with unspecified type of pneumonia | Non-infectious |
| J12.1 | Respiratory syncytial virus pneumonia | Non-infectious |
| J12.2 | Parainfluenza virus pneumonia | Non-infectious |
| J12.3 | Human metapneumovirus pneumonia | Non-infectious |
| J12.89 | Other viral pneumonia | Non-infectious |
| J12.9 | Viral pneumonia, unspecified | Non-infectious |
| J15.0 | Pneumonia due to Klebsiella pneumoniae | Pulmonary |
| J15.1 | Pneumonia due to Pseudomonas | Pulmonary |
| J15.211 | Pneumonia due to methicillin susceptible Staphylococcus aureus | Pulmonary |
| J15.4 | Pneumonia due to other streptococci | Pulmonary |
| J15.6 | Pneumonia due to other gram-negative bacteria | Pulmonary |
| J15.7 | Pneumonia due to Mycoplasma pneumoniae | Pulmonary |
| J15.8 | Pneumonia due to other specified bacteria | Pulmonary |
| J15.9 | Unspecified bacterial pneumonia | Pulmonary |
| J16.8 | Pneumonia due to other specified infectious organisms | Pulmonary |
| J18.0 | Bronchopneumonia, unspecified organism | Pulmonary |
| J18.1 | Lobar pneumonia, unspecified organism | Pulmonary |
| J18.8 | Other pneumonia, unspecified organism | Pulmonary |
| J18.9 | Pneumonia, unspecified organism | Pulmonary |
| J20.4 | Acute bronchitis due to parainfluenza virus | Non-infectious |
| J20.6 | Acute bronchitis due to Rhinovirus | Non-infectious |
| J20.9 | Acute bronchitis, unspecified | Non-infectious |
| J21.0 | Acute bronchiolitis due to respiratory syncytial virus | Non-infectious |
| J32.3 | Chronic sphenoidal sinusitis | Other |
| J32.8 | Other chronic sinusitis | Other |
| J32.9 | Chronic sinusitis, unspecified | Other |
| J33.8 | Other polyp of sinus | Non-infectious |
| J34.0 | Abscess, furuncle and carbuncle of nose | SSTI |
| J36 | Peritonsillar abscess | Other |
| J38.01 | Paralysis of vocal cords and larynx, unilateral | Non-infectious |
| J38.02 | Paralysis of vocal cords and larynx, bilateral | Non-infectious |
| J38.6 | Stenosis of larynx | Non-infectious |
| J39.0 | Retropharyngeal and parapharyngeal abscess | Other |
| J39.1 | Other abscess of pharynx | Other |
| J39.2 | Other diseases of pharynx | Non-infectious |
| J39.8 | Other specified diseases of upper respiratory tract | Non-infectious |
| J40 | Bronchitis, not specified as acute or chronic | Non-infectious |
| J43.2 | Centrilobular emphysema | Non-infectious |
| J43.9 | Emphysema, unspecified | Non-infectious |
| J44.0 | Chronic obstructive pulmonary disease with (acute) lower respiratory infection | Pulmonary |
| J44.1 | Chronic obstructive pulmonary disease with (acute) exacerbation | Pulmonary |
| J44.9 | Chronic obstructive pulmonary disease, unspecified | Non-infectious |
| J45.21 | Mild intermittent asthma with (acute) exacerbation | Non-infectious |
| J45.31 | Mild persistent asthma with (acute) exacerbation | Non-infectious |
| J45.41 | Moderate persistent asthma with (acute) exacerbation | Non-infectious |
| J45.51 | Severe persistent asthma with (acute) exacerbation | Non-infectious |
| J45.901 | Unspecified asthma with (acute) exacerbation | Non-infectious |
| J47.0 | Bronchiectasis with acute lower respiratory infection | Pulmonary |
| J47.1 | Bronchiectasis with (acute) exacerbation | Pulmonary |
| J47.9 | Bronchiectasis, uncomplicated | Non-infectious |
| J67.8 | Hypersensitivity pneumonitis due to other organic dusts | Non-infectious |
| J67.9 | Hypersensitivity pneumonitis due to unspecified organic dust | Non-infectious |
| J69.0 | Pneumonitis due to inhalation of food and vomit | Non-infectious |
| J70.2 | Acute drug-induced interstitial lung disorders | Non-infectious |
| J70.4 | Drug-induced interstitial lung disorders, unspecified | Non-infectious |
| J80 | Acute respiratory distress syndrome | Non-infectious |
| J81.0 | Acute pulmonary edema | Non-infectious |
| J82.81 | Chronic eosinophilic pneumonia | Non-infectious |
| J84.10 | Pulmonary fibrosis, unspecified | Non-infectious |
| J84.112 | Idiopathic pulmonary fibrosis | Non-infectious |
| J84.113 | Idiopathic non-specific interstitial pneumonitis | Non-infectious |
| J84.114 | Acute interstitial pneumonitis | Non-infectious |
| J84.116 | Cryptogenic organizing pneumonia | Non-infectious |
| J84.89 | Other specified interstitial pulmonary diseases | Non-infectious |
| J84.9 | Interstitial pulmonary disease, unspecified | Non-infectious |
| J85.0 | Gangrene and necrosis of lung | Pulmonary |
| J85.1 | Abscess of lung with pneumonia | Pulmonary |
| J85.2 | Abscess of lung without pneumonia | Pulmonary |
| J85.3 | Abscess of mediastinum | Other |
| J86.0 | Pyothorax with fistula | Non-infectious |
| J86.9 | Pyothorax without fistula | Pulmonary |
| J90 | Pleural effusion, not elsewhere classified | Non-infectious |
| J93.83 | Other pneumothorax | Non-infectious |
| J93.9 | Pneumothorax, unspecified | Non-infectious |
| J94.2 | Hemothorax | Non-infectious |
| J94.8 | Other specified pleural conditions | Non-infectious |
| J95.01 | Hemorrhage from tracheostomy stoma | Non-infectious |
| J95.02 | Infection of tracheostomy stoma | SSTI |
| J95.03 | Malfunction of tracheostomy stoma | Non-infectious |
| J95.09 | Other tracheostomy complication | Non-infectious |
| J95.5 | Postprocedural subglottic stenosis | Non-infectious |
| J95.61 | Intraoperative hemorrhage and hematoma of a respiratory system organ or structure complicating a respiratory system procedure | Non-infectious |
| J95.811 | Postprocedural pneumothorax | Non-infectious |
| J95.851 | Ventilator associated pneumonia | Pulmonary |
| J95.859 | Other complication of respirator (ventilator) | Non-infectious |
| J95.860 | Postprocedural hematoma of a respiratory system organ or structure following a respiratory system procedure | Non-infectious |
| J95.89 | Other postprocedural complications and disorders of respiratory system, not elsewhere classified | Non-infectious |
| J96.00 | Acute respiratory failure, unspecified whether with hypoxia or hypercapnia | Non-infectious |
| J96.01 | Acute respiratory failure with hypoxia | Non-infectious |
| J96.02 | Acute respiratory failure with hypercapnia | Non-infectious |
| J96.11 | Chronic respiratory failure with hypoxia | Non-infectious |
| J96.21 | Acute and chronic respiratory failure with hypoxia | Non-infectious |
| J96.22 | Acute and chronic respiratory failure with hypercapnia | Non-infectious |
| J96.91 | Respiratory failure, unspecified with hypoxia | Non-infectious |
| J98.09 | Other diseases of bronchus, not elsewhere classified | Non-infectious |
| J98.2 | Interstitial emphysema | Non-infectious |
| J98.4 | Other disorders of lung | Non-infectious |
| J98.6 | Disorders of diaphragm | Non-infectious |
| K02.9 | Dental caries, unspecified | Non-infectious |
| K04.1 | Necrosis of pulp | Non-infectious |
| K04.7 | Periapical abscess without sinus | Other |
| K05.219 | Aggressive periodontitis, localized, unspecified severity | Other |
| K05.6 | Periodontal disease, unspecified | Non-infectious |
| K11.20 | Sialoadenitis, unspecified | Other |
| K11.21 | Acute sialoadenitis | Other |
| K11.3 | Abscess of salivary gland | Other |
| K12.2 | Cellulitis and abscess of mouth | Other |
| K12.30 | Oral mucositis (ulcerative), unspecified | Non-infectious |
| K12.31 | Oral mucositis (ulcerative) due to antineoplastic therapy | Non-infectious |
| K12.33 | Oral mucositis (ulcerative) due to radiation | Non-infectious |
| K13.0 | Diseases of lips | Non-infectious |
| K14.0 | Glossitis | Non-infectious |
| K20.0 | Eosinophilic esophagitis | Non-infectious |
| K20.80 | Other esophagitis without bleeding | Non-infectious |
| K20.90 | Esophagitis, unspecified without bleeding | Non-infectious |
| K20.91 | Esophagitis, unspecified with bleeding | Non-infectious |
| K21.00 | Gastro-esophageal reflux disease with esophagitis, without bleeding | Non-infectious |
| K21.01 | Gastro-esophageal reflux disease with esophagitis, with bleeding | Non-infectious |
| K21.9 | Gastro-esophageal reflux disease without esophagitis | Non-infectious |
| K22.0 | Achalasia of cardia | Non-infectious |
| K22.10 | Ulcer of esophagus without bleeding | Non-infectious |
| K22.11 | Ulcer of esophagus with bleeding | Non-infectious |
| K22.2 | Esophageal obstruction | Non-infectious |
| K22.3 | Perforation of esophagus | Abdomen/pelvis |
| K22.4 | Dyskinesia of esophagus | Non-infectious |
| K22.5 | Diverticulum of esophagus, acquired | Non-infectious |
| K22.6 | Gastro-esophageal laceration-hemorrhage syndrome | Non-infectious |
| K22.711 | Barrett's esophagus with high grade dysplasia | Non-infectious |
| K25.0 | Acute gastric ulcer with hemorrhage | Non-infectious |
| K25.1 | Acute gastric ulcer with perforation | Non-infectious |
| K25.4 | Chronic or unspecified gastric ulcer with hemorrhage | Non-infectious |
| K25.5 | Chronic or unspecified gastric ulcer with perforation | Non-infectious |
| K25.9 | Gastric ulcer, unspecified as acute or chronic, without hemorrhage or perforation | Non-infectious |
| K26.4 | Chronic or unspecified duodenal ulcer with hemorrhage | Non-infectious |
| K26.5 | Chronic or unspecified duodenal ulcer with perforation | Non-infectious |
| K26.6 | Chronic or unspecified duodenal ulcer with both hemorrhage and perforation | Non-infectious |
| K26.9 | Duodenal ulcer, unspecified as acute or chronic, without hemorrhage or perforation | Non-infectious |
| K28.1 | Acute gastrojejunal ulcer with perforation | Non-infectious |
| K28.4 | Chronic or unspecified gastrojejunal ulcer with hemorrhage | Non-infectious |
| K28.5 | Chronic or unspecified gastrojejunal ulcer with perforation | Non-infectious |
| K29.00 | Acute gastritis without bleeding | Non-infectious |
| K29.41 | Chronic atrophic gastritis with bleeding | Non-infectious |
| K29.50 | Unspecified chronic gastritis without bleeding | Non-infectious |
| K29.51 | Unspecified chronic gastritis with bleeding | Non-infectious |
| K29.70 | Gastritis, unspecified, without bleeding | Non-infectious |
| K29.71 | Gastritis, unspecified, with bleeding | Non-infectious |
| K29.80 | Duodenitis without bleeding | Non-infectious |
| K30 | Functional dyspepsia | Non-infectious |
| K31.1 | Adult hypertrophic pyloric stenosis | Non-infectious |
| K31.5 | Obstruction of duodenum | Non-infectious |
| K31.6 | Fistula of stomach and duodenum | Non-infectious |
| K31.7 | Polyp of stomach and duodenum | Non-infectious |
| K31.811 | Angiodysplasia of stomach and duodenum with bleeding | Non-infectious |
| K31.84 | Gastroparesis | Non-infectious |
| K31.89 | Other diseases of stomach and duodenum | Non-infectious |
| K35.20 | Acute appendicitis with generalized peritonitis, without abscess | Abdomen/pelvis |
| K35.200 | Acute appendicitis with generalized peritonitis, without perforation or abscess | Abdomen/pelvis |
| K35.201 | Acute appendicitis with generalized peritonitis, with perforation, without abscess | Abdomen/pelvis |
| K35.21 | Acute appendicitis with generalized peritonitis, with abscess | Abdomen/pelvis |
| K35.30 | Acute appendicitis with localized peritonitis, without perforation or gangrene | Abdomen/pelvis |
| K35.31 | Acute appendicitis with localized peritonitis and gangrene, without perforation | Abdomen/pelvis |
| K35.32 | Acute appendicitis with perforation, localized peritonitis, and gangrene, without abscess | Abdomen/pelvis |
| K35.33 | Acute appendicitis with perforation, localized peritonitis, and gangrene, with abscess | Abdomen/pelvis |
| K35.80 | Unspecified acute appendicitis | Abdomen/pelvis |
| K35.890 | Other acute appendicitis without perforation or gangrene | Abdomen/pelvis |
| K35.891 | Other acute appendicitis without perforation, with gangrene | Abdomen/pelvis |
| K37 | Unspecified appendicitis | Abdomen/pelvis |
| K40.30 | Unilateral inguinal hernia, with obstruction, without gangrene, not specified as recurrent | Non-infectious |
| K40.31 | Unilateral inguinal hernia, with obstruction, without gangrene, recurrent | Non-infectious |
| K41.30 | Unilateral femoral hernia, with obstruction, without gangrene, not specified as recurrent | Non-infectious |
| K42.0 | Umbilical hernia with obstruction, without gangrene | Non-infectious |
| K43.0 | Incisional hernia with obstruction, without gangrene | Non-infectious |
| K43.2 | Incisional hernia without obstruction or gangrene | Non-infectious |
| K43.3 | Parastomal hernia with obstruction, without gangrene | Non-infectious |
| K43.5 | Parastomal hernia without obstruction or gangrene | Non-infectious |
| K43.6 | Other and unspecified ventral hernia with obstruction, without gangrene | Non-infectious |
| K43.9 | Ventral hernia without obstruction or gangrene | Non-infectious |
| K44.0 | Diaphragmatic hernia with obstruction, without gangrene | Non-infectious |
| K44.9 | Diaphragmatic hernia without obstruction or gangrene | Non-infectious |
| K46.0 | Unspecified abdominal hernia with obstruction, without gangrene | Non-infectious |
| K50.00 | Crohn's disease of small intestine without complications | Non-infectious |
| K50.011 | Crohn's disease of small intestine with rectal bleeding | Non-infectious |
| K50.012 | Crohn's disease of small intestine with intestinal obstruction | Non-infectious |
| K50.013 | Crohn's disease of small intestine with fistula | Non-infectious |
| K50.014 | Crohn's disease of small intestine with abscess | Abdomen/pelvis |
| K50.111 | Crohn's disease of large intestine with rectal bleeding | Non-infectious |
| K50.112 | Crohn's disease of large intestine with intestinal obstruction | Non-infectious |
| K50.113 | Crohn's disease of large intestine with fistula | Non-infectious |
| K50.114 | Crohn's disease of large intestine with abscess | Abdomen/pelvis |
| K50.118 | Crohn's disease of large intestine with other complication | Non-infectious |
| K50.80 | Crohn's disease of both small and large intestine without complications | Non-infectious |
| K50.812 | Crohn's disease of both small and large intestine with intestinal obstruction | Non-infectious |
| K50.813 | Crohn's disease of both small and large intestine with fistula | Non-infectious |
| K50.814 | Crohn's disease of both small and large intestine with abscess | Abdomen/pelvis |
| K50.90 | Crohn's disease, unspecified, without complications | Non-infectious |
| K50.914 | Crohn's disease, unspecified, with abscess | Abdomen/pelvis |
| K51.00 | Ulcerative (chronic) pancolitis without complications | Non-infectious |
| K51.011 | Ulcerative (chronic) pancolitis with rectal bleeding | Non-infectious |
| K51.018 | Ulcerative (chronic) pancolitis with other complication | Non-infectious |
| K51.20 | Ulcerative (chronic) proctitis without complications | Non-infectious |
| K51.30 | Ulcerative (chronic) rectosigmoiditis without complications | Non-infectious |
| K51.311 | Ulcerative (chronic) rectosigmoiditis with rectal bleeding | Non-infectious |
| K51.519 | Left sided colitis with unspecified complications | Abdomen/pelvis |
| K51.80 | Other ulcerative colitis without complications | Non-infectious |
| K51.814 | Other ulcerative colitis with abscess | Abdomen/pelvis |
| K51.90 | Ulcerative colitis, unspecified, without complications | Non-infectious |
| K51.914 | Ulcerative colitis, unspecified with abscess | Abdomen/pelvis |
| K51.918 | Ulcerative colitis, unspecified with other complication | Non-infectious |
| K51.919 | Ulcerative colitis, unspecified with unspecified complications | Non-infectious |
| K52.1 | Toxic gastroenteritis and colitis | Non-infectious |
| K52.89 | Other specified noninfective gastroenteritis and colitis | Non-infectious |
| K52.9 | Noninfective gastroenteritis and colitis, unspecified | Abdomen/pelvis |
| K55.021 | Focal (segmental) acute infarction of small intestine | Non-infectious |
| K55.031 | Focal (segmental) acute (reversible) ischemia of large intestine | Non-infectious |
| K55.039 | Acute (reversible) ischemia of large intestine, extent unspecified | Non-infectious |
| K55.041 | Focal (segmental) acute infarction of large intestine | Non-infectious |
| K55.059 | Acute (reversible) ischemia of intestine, part and extent unspecified | Non-infectious |
| K55.1 | Chronic vascular disorders of intestine | Non-infectious |
| K55.21 | Angiodysplasia of colon with hemorrhage | Non-infectious |
| K55.8 | Other vascular disorders of intestine | Non-infectious |
| K55.9 | Vascular disorder of intestine, unspecified | Non-infectious |
| K56.1 | Intussusception | Non-infectious |
| K56.2 | Volvulus | Non-infectious |
| K56.3 | Gallstone ileus | Non-infectious |
| K56.41 | Fecal impaction | Non-infectious |
| K56.49 | Other impaction of intestine | Non-infectious |
| K56.50 | Intestinal adhesions (bands), unspecified as to partial versus complete obstruction | Non-infectious |
| K56.51 | Intestinal adhesions (bands), with partial obstruction | Non-infectious |
| K56.52 | Intestinal adhesions (bands) with complete obstruction | Non-infectious |
| K56.600 | Partial intestinal obstruction, unspecified as to cause | Non-infectious |
| K56.609 | Unspecified intestinal obstruction, unspecified as to partial versus complete obstruction | Non-infectious |
| K56.690 | Other partial intestinal obstruction | Non-infectious |
| K56.699 | Other intestinal obstruction unspecified as to partial versus complete obstruction | Non-infectious |
| K56.7 | Ileus, unspecified | Non-infectious |
| K57.00 | Diverticulitis of small intestine with perforation and abscess without bleeding | Abdomen/pelvis |
| K57.10 | Diverticulosis of small intestine without perforation or abscess without bleeding | Non-infectious |
| K57.11 | Diverticulosis of small intestine without perforation or abscess with bleeding | Non-infectious |
| K57.12 | Diverticulitis of small intestine without perforation or abscess without bleeding | Abdomen/pelvis |
| K57.20 | Diverticulitis of large intestine with perforation and abscess without bleeding | Abdomen/pelvis |
| K57.21 | Diverticulitis of large intestine with perforation and abscess with bleeding | Abdomen/pelvis |
| K57.31 | Diverticulosis of large intestine without perforation or abscess with bleeding | Non-infectious |
| K57.32 | Diverticulitis of large intestine without perforation or abscess without bleeding | Abdomen/pelvis |
| K57.33 | Diverticulitis of large intestine without perforation or abscess with bleeding | Abdomen/pelvis |
| K57.52 | Diverticulitis of both small and large intestine without perforation or abscess without bleeding | Abdomen/pelvis |
| K57.80 | Diverticulitis of intestine, part unspecified, with perforation and abscess without bleeding | Abdomen/pelvis |
| K57.91 | Diverticulosis of intestine, part unspecified, without perforation or abscess with bleeding | Non-infectious |
| K57.93 | Diverticulitis of intestine, part unspecified, without perforation or abscess with bleeding | Abdomen/pelvis |
| K58.0 | Irritable bowel syndrome with diarrhea | Non-infectious |
| K58.2 | Mixed irritable bowel syndrome | Non-infectious |
| K59.00 | Constipation, unspecified | Non-infectious |
| K59.03 | Drug induced constipation | Non-infectious |
| K59.09 | Other constipation | Non-infectious |
| K59.31 | Toxic megacolon | Non-infectious |
| K59.81 | Ogilvie syndrome | Non-infectious |
| K59.89 | Other specified functional intestinal disorders | Non-infectious |
| K60.2 | Anal fissure, unspecified | Non-infectious |
| K60.5 | Anorectal fistula | Non-infectious |
| K61.0 | Anal abscess | Abdomen/pelvis |
| K61.1 | Rectal abscess | Abdomen/pelvis |
| K61.2 | Anorectal abscess | Abdomen/pelvis |
| K61.31 | Horseshoe abscess | Abdomen/pelvis |
| K61.39 | Other ischiorectal abscess | Abdomen/pelvis |
| K61.4 | Intrasphincteric abscess | Abdomen/pelvis |
| K61.5 | Supralevator abscess | Abdomen/pelvis |
| K62.3 | Rectal prolapse | Abdomen/pelvis |
| K62.4 | Stenosis of anus and rectum | Non-infectious |
| K62.6 | Ulcer of anus and rectum | Non-infectious |
| K62.7 | Radiation proctitis | Non-infectious |
| K62.89 | Other specified diseases of anus and rectum | Non-infectious |
| K63.0 | Abscess of intestine | Abdomen/pelvis |
| K63.1 | Perforation of intestine (nontraumatic) | Abdomen/pelvis |
| K63.2 | Fistula of intestine | Non-infectious |
| K63.3 | Ulcer of intestine | Non-infectious |
| K63.8219 | Small intestinal bacterial overgrowth, unspecified | Abdomen/pelvis |
| K63.829 | Intestinal methanogen overgrowth, unspecified | Non-infectious |
| K63.89 | Other specified diseases of intestine | Non-infectious |
| K64.4 | Residual hemorrhoidal skin tags | Non-infectious |
| K64.8 | Other hemorrhoids | Non-infectious |
| K65.1 | Peritoneal abscess | Abdomen/pelvis |
| K65.2 | Spontaneous bacterial peritonitis | Abdomen/pelvis |
| K65.4 | Sclerosing mesenteritis | Non-infectious |
| K65.8 | Other peritonitis | Abdomen/pelvis |
| K65.9 | Peritonitis, unspecified | Abdomen/pelvis |
| K66.1 | Hemoperitoneum | Non-infectious |
| K68.11 | Postprocedural retroperitoneal abscess | Other |
| K68.12 | Psoas muscle abscess | Other |
| K68.19 | Other retroperitoneal abscess | Other |
| K68.9 | Other disorders of retroperitoneum | Non-infectious |
| K70.10 | Alcoholic hepatitis without ascites | Non-infectious |
| K70.11 | Alcoholic hepatitis with ascites | Non-infectious |
| K70.30 | Alcoholic cirrhosis of liver without ascites | Non-infectious |
| K70.31 | Alcoholic cirrhosis of liver with ascites | Non-infectious |
| K70.40 | Alcoholic hepatic failure without coma | Non-infectious |
| K71.0 | Toxic liver disease with cholestasis | Non-infectious |
| K71.8 | Toxic liver disease with other disorders of liver | Non-infectious |
| K72.00 | Acute and subacute hepatic failure without coma | Non-infectious |
| K72.10 | Chronic hepatic failure without coma | Non-infectious |
| K72.90 | Hepatic failure, unspecified without coma | Non-infectious |
| K74.3 | Primary biliary cirrhosis | Non-infectious |
| K74.5 | Biliary cirrhosis, unspecified | Non-infectious |
| K74.60 | Unspecified cirrhosis of liver | Non-infectious |
| K74.69 | Other cirrhosis of liver | Non-infectious |
| K75.0 | Abscess of liver | Abdomen/pelvis |
| K75.4 | Autoimmune hepatitis | Non-infectious |
| K75.81 | Nonalcoholic steatohepatitis (NASH) | Non-infectious |
| K75.89 | Other specified inflammatory liver diseases | Non-infectious |
| K76.6 | Portal hypertension | Non-infectious |
| K76.7 | Hepatorenal syndrome | Non-infectious |
| K76.82 | Hepatic encephalopathy | Non-infectious |
| K76.89 | Other specified diseases of liver | Non-infectious |
| K80.00 | Calculus of gallbladder with acute cholecystitis without obstruction | Abdomen/pelvis |
| K80.01 | Calculus of gallbladder with acute cholecystitis with obstruction | Abdomen/pelvis |
| K80.10 | Calculus of gallbladder with chronic cholecystitis without obstruction | Abdomen/pelvis |
| K80.12 | Calculus of gallbladder with acute and chronic cholecystitis without obstruction | Abdomen/pelvis |
| K80.13 | Calculus of gallbladder with acute and chronic cholecystitis with obstruction | Abdomen/pelvis |
| K80.20 | Calculus of gallbladder without cholecystitis without obstruction | Non-infectious |
| K80.21 | Calculus of gallbladder without cholecystitis with obstruction | Non-infectious |
| K80.30 | Calculus of bile duct with cholangitis, unspecified, without obstruction | Abdomen/pelvis |
| K80.31 | Calculus of bile duct with cholangitis, unspecified, with obstruction | Abdomen/pelvis |
| K80.32 | Calculus of bile duct with acute cholangitis without obstruction | Abdomen/pelvis |
| K80.35 | Calculus of bile duct with chronic cholangitis with obstruction | Abdomen/pelvis |
| K80.36 | Calculus of bile duct with acute and chronic cholangitis without obstruction | Abdomen/pelvis |
| K80.41 | Calculus of bile duct with cholecystitis, unspecified, with obstruction | Abdomen/pelvis |
| K80.42 | Calculus of bile duct with acute cholecystitis without obstruction | Abdomen/pelvis |
| K80.43 | Calculus of bile duct with acute cholecystitis with obstruction | Abdomen/pelvis |
| K80.46 | Calculus of bile duct with acute and chronic cholecystitis without obstruction | Abdomen/pelvis |
| K80.50 | Calculus of bile duct without cholangitis or cholecystitis without obstruction | Abdomen/pelvis |
| K80.51 | Calculus of bile duct without cholangitis or cholecystitis with obstruction | Non-infectious |
| K80.62 | Calculus of gallbladder and bile duct with acute cholecystitis without obstruction | Abdomen/pelvis |
| K80.63 | Calculus of gallbladder and bile duct with acute cholecystitis with obstruction | Abdomen/pelvis |
| K80.64 | Calculus of gallbladder and bile duct with chronic cholecystitis without obstruction | Abdomen/pelvis |
| K80.66 | Calculus of gallbladder and bile duct with acute and chronic cholecystitis without obstruction | Abdomen/pelvis |
| K80.67 | Calculus of gallbladder and bile duct with acute and chronic cholecystitis with obstruction | Abdomen/pelvis |
| K80.70 | Calculus of gallbladder and bile duct without cholecystitis without obstruction | Non-infectious |
| K80.71 | Calculus of gallbladder and bile duct without cholecystitis with obstruction | Non-infectious |
| K81.0 | Acute cholecystitis | Abdomen/pelvis |
| K81.1 | Chronic cholecystitis | Abdomen/pelvis |
| K81.2 | Acute cholecystitis with chronic cholecystitis | Abdomen/pelvis |
| K81.9 | Cholecystitis, unspecified | Abdomen/pelvis |
| K82.8 | Other specified diseases of gallbladder | Non-infectious |
| K83.01 | Primary sclerosing cholangitis | Non-infectious |
| K83.09 | Other cholangitis | Abdomen/pelvis |
| K83.1 | Obstruction of bile duct | Non-infectious |
| K83.3 | Fistula of bile duct | Non-infectious |
| K85.10 | Biliary acute pancreatitis without necrosis or infection | Non-infectious |
| K85.11 | Biliary acute pancreatitis with uninfected necrosis | Non-infectious |
| K85.12 | Biliary acute pancreatitis with infected necrosis | Abdomen/pelvis |
| K85.20 | Alcohol induced acute pancreatitis without necrosis or infection | Non-infectious |
| K85.21 | Alcohol induced acute pancreatitis with uninfected necrosis | Non-infectious |
| K85.80 | Other acute pancreatitis without necrosis or infection | Non-infectious |
| K85.81 | Other acute pancreatitis with uninfected necrosis | Non-infectious |
| K85.82 | Other acute pancreatitis with infected necrosis | Abdomen/pelvis |
| K85.90 | Acute pancreatitis without necrosis or infection, unspecified | Non-infectious |
| K85.91 | Acute pancreatitis with uninfected necrosis, unspecified | Non-infectious |
| K85.92 | Acute pancreatitis with infected necrosis, unspecified | Abdomen/pelvis |
| K86.1 | Other chronic pancreatitis | Non-infectious |
| K86.2 | Cyst of pancreas | Non-infectious |
| K86.3 | Pseudocyst of pancreas | Non-infectious |
| K86.81 | Exocrine pancreatic insufficiency | Non-infectious |
| K86.89 | Other specified diseases of pancreas | Non-infectious |
| K86.9 | Disease of pancreas, unspecified | Non-infectious |
| K90.89 | Other intestinal malabsorption | Non-infectious |
| K91.30 | Postprocedural intestinal obstruction, unspecified as to partial versus complete | Non-infectious |
| K91.31 | Postprocedural partial intestinal obstruction | Non-infectious |
| K91.32 | Postprocedural complete intestinal obstruction | Non-infectious |
| K91.71 | Accidental puncture and laceration of a digestive system organ or structure during a digestive system procedure | Abdomen/pelvis |
| K91.72 | Accidental puncture and laceration of a digestive system organ or structure during other procedure | Abdomen/pelvis |
| K91.81 | Other intraoperative complications of digestive system | Non-infectious |
| K91.840 | Postprocedural hemorrhage of a digestive system organ or structure following a digestive system procedure | Non-infectious |
| K91.841 | Postprocedural hemorrhage of a digestive system organ or structure following other procedure | Non-infectious |
| K91.850 | Pouchitis | Abdomen/pelvis |
| K91.86 | Retained cholelithiasis following cholecystectomy | Non-infectious |
| K91.871 | Postprocedural hematoma of a digestive system organ or structure following other procedure | Non-infectious |
| K91.872 | Postprocedural seroma of a digestive system organ or structure following a digestive system procedure | Non-infectious |
| K91.873 | Postprocedural seroma of a digestive system organ or structure following other procedure | Non-infectious |
| K91.89 | Other postprocedural complications and disorders of digestive system | Non-infectious |
| K92.0 | Hematemesis | Non-infectious |
| K92.1 | Melena | Non-infectious |
| K92.2 | Gastrointestinal hemorrhage, unspecified | Non-infectious |
| K94.02 | Colostomy infection | Abdomen/pelvis |
| K94.03 | Colostomy malfunction | Non-infectious |
| K94.09 | Other complications of colostomy | Non-infectious |
| K94.12 | Enterostomy infection | Abdomen/pelvis |
| K94.13 | Enterostomy malfunction | Non-infectious |
| K94.19 | Other complications of enterostomy | Non-infectious |
| K94.21 | Gastrostomy hemorrhage | Non-infectious |
| K94.22 | Gastrostomy infection | Abdomen/pelvis |
| K94.23 | Gastrostomy malfunction | Non-infectious |
| L01.02 | Bockhart's impetigo | Non-infectious |
| L02.01 | Cutaneous abscess of face | SSTI |
| L02.11 | Cutaneous abscess of neck | SSTI |
| L02.211 | Cutaneous abscess of abdominal wall | SSTI |
| L02.212 | Cutaneous abscess of back (any part, except buttock) | SSTI |
| L02.213 | Cutaneous abscess of chest wall | SSTI |
| L02.214 | Cutaneous abscess of groin | SSTI |
| L02.215 | Cutaneous abscess of perineum | SSTI |
| L02.221 | Furuncle of abdominal wall | SSTi |
| L02.31 | Cutaneous abscess of buttock | SSTI |
| L02.411 | Cutaneous abscess of right axilla | SSTI |
| L02.412 | Cutaneous abscess of left axilla | SSTI |
| L02.413 | Cutaneous abscess of right upper limb | SSTI |
| L02.414 | Cutaneous abscess of left upper limb | SSTI |
| L02.415 | Cutaneous abscess of right lower limb | SSTI |
| L02.416 | Cutaneous abscess of left lower limb | SSTI |
| L02.511 | Cutaneous abscess of right hand | SSTI |
| L02.512 | Cutaneous abscess of left hand | SSTI |
| L02.811 | Cutaneous abscess of head (any part, except face) | SSTI |
| L02.821 | Furuncle of head (any part, except face) | SSTI |
| L03.011 | Cellulitis of right finger | SSTI |
| L03.012 | Cellulitis of left finger | SSTI |
| L03.031 | Cellulitis of right toe | SSTI |
| L03.032 | Cellulitis of left toe | SSTI |
| L03.111 | Cellulitis of right axilla | SSTI |
| L03.112 | Cellulitis of left axilla | SSTI |
| L03.113 | Cellulitis of right upper limb | SSTI |
| L03.114 | Cellulitis of left upper limb | SSTI |
| L03.115 | Cellulitis of right lower limb | SSTI |
| L03.116 | Cellulitis of left lower limb | SSTI |
| L03.211 | Cellulitis of face | SSTI |
| L03.213 | Periorbital cellulitis | SSTI |
| L03.221 | Cellulitis of neck | SSTI |
| L03.311 | Cellulitis of abdominal wall | SSTI |
| L03.312 | Cellulitis of back (any part except buttock) | SSTI |
| L03.313 | Cellulitis of chest wall | SSTI |
| L03.314 | Cellulitis of groin | SSTI |
| L03.317 | Cellulitis of buttock | SSTI |
| L03.811 | Cellulitis of head (any part, except face) | SSTI |
| L04.0 | Acute lymphadenitis of face, head and neck | SSTI |
| L05.01 | Pilonidal cyst with abscess | SSTI |
| L08.0 | Pyoderma | SSTI |
| L08.89 | Other specified local infections of the skin and subcutaneous tissue | SSTI |
| L08.9 | Local infection of the skin and subcutaneous tissue, unspecified | SSTI |
| L10.2 | Pemphigus foliaceous | Non-infectious |
| L12.0 | Bullous pemphigoid | Non-infectious |
| L12.8 | Other pemphigoid | Non-infectious |
| L20.9 | Atopic dermatitis, unspecified | Non-infectious |
| L21.9 | Seborrheic dermatitis, unspecified | Non-infectious |
| L23.3 | Allergic contact dermatitis due to drugs in contact with skin | Non-infectious |
| L23.9 | Allergic contact dermatitis, unspecified cause | Non-infectious |
| L26 | Exfoliative dermatitis | Non-infectious |
| L27.0 | Generalized skin eruption due to drugs and medicaments taken internally | Non-infectious |
| L29.9 | Pruritus, unspecified | Non-infectious |
| L30.8 | Other specified dermatitis | Non-infectious |
| L30.9 | Dermatitis, unspecified | Non-infectious |
| L43.8 | Other lichen planus | Non-infectious |
| L50.0 | Allergic urticaria | Non-infectious |
| L73.2 | Hidradenitis suppurativa | Non-infectious |
| L73.8 | Other specified follicular disorders | Non-infectious |
| L76.22 | Postprocedural hemorrhage of skin and subcutaneous tissue following other procedure | Non-infectious |
| L76.32 | Postprocedural hematoma of skin and subcutaneous tissue following other procedure | Non-infectious |
| L76.33 | Postprocedural seroma of skin and subcutaneous tissue following a dermatologic procedure | Non-infectious |
| L76.34 | Postprocedural seroma of skin and subcutaneous tissue following other procedure | Non-infectious |
| L76.82 | Other postprocedural complications of skin and subcutaneous tissue | Non-infectious |
| L88 | Pyoderma gangrenosum | Non-infectious |
| L89.152 | Pressure ulcer of sacral region, stage 2 | Non-infectious |
| L89.153 | Pressure ulcer of sacral region, stage 3 | Non-infectious |
| L89.154 | Pressure ulcer of sacral region, stage 4 | Non-infectious |
| L89.159 | Pressure ulcer of sacral region, unspecified stage | Non-infectious |
| L89.224 | Pressure ulcer of left hip, stage 4 | Non-infectious |
| L89.226 | Pressure-induced deep tissue damage of left hip | Non-infectious |
| L89.310 | Pressure ulcer of right buttock, unstageable | Non-infectious |
| L89.314 | Pressure ulcer of right buttock, stage 4 | Non-infectious |
| L89.324 | Pressure ulcer of left buttock, stage 4 | Non-infectious |
| L89.894 | Pressure ulcer of other site, stage 4 | Non-infectious |
| L95.0 | Livedoid vasculitis | Non-infectious |
| L97.319 | Non-pressure chronic ulcer of right ankle with unspecified severity | Non-infectious |
| L97.429 | Non-pressure chronic ulcer of left heel and midfoot with unspecified severity | Non-infectious |
| L97.519 | Non-pressure chronic ulcer of other part of right foot with unspecified severity | Non-infectious |
| L97.819 | Non-pressure chronic ulcer of other part of right lower leg with unspecified severity | Non-infectious |
| L97.909 | Non-pressure chronic ulcer of unspecified part of unspecified lower leg with unspecified severity | Non-infectious |
| L97.919 | Non-pressure chronic ulcer of unspecified part of right lower leg with unspecified severity | Non-infectious |
| L98.2 | Febrile neutrophilic dermatosis (sweet) | Non-infectious |
| L98.419 | Non-pressure chronic ulcer of buttock with unspecified severity | Non-infectious |
| L98.499 | Non-pressure chronic ulcer of skin of other sites with unspecified severity | Non-infectious |
| L98.8 | Other specified disorders of the skin and subcutaneous tissue | Non-infectious |
| L98.9 | Disorder of the skin and subcutaneous tissue, unspecified | Non-infectious |
| M00.051 | Staphylococcal arthritis, right hip | bone/joint infections |
| M00.261 | Other streptococcal arthritis, right knee | bone/joint infections |
| M00.262 | Other streptococcal arthritis, left knee | bone/joint infections |
| M00.842 | Arthritis due to other bacteria, left hand | bone/joint infections |
| M00.861 | Arthritis due to other bacteria, right knee | bone/joint infections |
| M00.9 | Pyogenic arthritis, unspecified | bone/joint infections |
| M05.841 | Other rheumatoid arthritis with rheumatoid factor of right hand | Non-infectious |
| M05.9 | Rheumatoid arthritis with rheumatoid factor, unspecified | Non-infectious |
| M06.9 | Rheumatoid arthritis, unspecified | Non-infectious |
| M10.062 | Idiopathic gout, left knee | Non-infectious |
| M10.9 | Gout, unspecified | Non-infectious |
| M11.231 | Other chondrocalcinosis, right wrist | Non-infectious |
| M11.262 | Other chondrocalcinosis, left knee | Non-infectious |
| M13.0 | Polyarthritis, unspecified | Non-infectious |
| M16.0 | Bilateral primary osteoarthritis of hip | Non-infectious |
| M16.11 | Unilateral primary osteoarthritis, right hip | Non-infectious |
| M16.12 | Unilateral primary osteoarthritis, left hip | Non-infectious |
| M17.0 | Bilateral primary osteoarthritis of knee | Non-infectious |
| M17.11 | Unilateral primary osteoarthritis, right knee | Non-infectious |
| M17.12 | Unilateral primary osteoarthritis, left knee | Non-infectious |
| M19.011 | Primary osteoarthritis, right shoulder | Non-infectious |
| M19.012 | Primary osteoarthritis, left shoulder | Non-infectious |
| M19.122 | Post-traumatic osteoarthritis, left elbow | Non-infectious |
| M1A.9XX1 | Chronic gout, unspecified, with tophus (tophi) | Non-infectious |
| M21.852 | Other specified acquired deformities of left thigh | Non-infectious |
| M25.062 | Hemarthrosis, left knee | Non-infectious |
| M25.461 | Effusion, right knee | Non-infectious |
| M25.512 | Pain in left shoulder | Non-infectious |
| M25.552 | Pain in left hip | Non-infectious |
| M26.01 | Maxillary hyperplasia | Non-infectious |
| M26.02 | Maxillary hypoplasia | Non-infectious |
| M26.212 | Malocclusion, Angle's class II | Non-infectious |
| M27.2 | Inflammatory conditions of jaws | Non-infectious |
| M31.0 | Hypersensitivity angiitis | Non-infectious |
| M31.10 | Thrombotic microangiopathy, unspecified | Non-infectious |
| M31.30 | Wegener's granulomatosis without renal involvement | Non-infectious |
| M32.13 | Lung involvement in systemic lupus erythematosus | Non-infectious |
| M32.14 | Glomerular disease in systemic lupus erythematosus | Non-infectious |
| M32.19 | Other organ or system involvement in systemic lupus erythematosus | Non-infectious |
| M32.9 | Systemic lupus erythematosus, unspecified | Non-infectious |
| M34.1 | Cr(e)st syndrome | Non-infectious |
| M34.81 | Systemic sclerosis with lung involvement | Non-infectious |
| M34.9 | Systemic sclerosis, unspecified | Non-infectious |
| M35.3 | Polymyalgia rheumatica | Non-infectious |
| M35.81 | Multisystem inflammatory syndrome | Non-infectious |
| M35.89 | Other specified systemic involvement of connective tissue | Non-infectious |
| M40.204 | Unspecified kyphosis, thoracic region | Non-infectious |
| M40.292 | Other kyphosis, cervical region | Non-infectious |
| M43.12 | Spondylolisthesis, cervical region | Non-infectious |
| M43.16 | Spondylolisthesis, lumbar region | Non-infectious |
| M43.17 | Spondylolisthesis, lumbosacral region | Non-infectious |
| M46.22 | Osteomyelitis of vertebra, cervical region | bone/joint infections |
| M46.26 | Osteomyelitis of vertebra, lumbar region | bone/joint infections |
| M46.28 | Osteomyelitis of vertebra, sacral and sacrococcygeal region | bone/joint infections |
| M46.39 | Infection of intervertebral disc (pyogenic), multiple sites in spine | bone/joint infections |
| M46.52 | Other infective spondylopathies, cervical region | bone/joint infections |
| M47.12 | Other spondylosis with myelopathy, cervical region | Non-infectious |
| M47.26 | Other spondylosis with radiculopathy, lumbar region | Non-infectious |
| M47.815 | Spondylosis without myelopathy or radiculopathy, thoracolumbar region | Non-infectious |
| M48.02 | Spinal stenosis, cervical region | Non-infectious |
| M48.061 | Spinal stenosis, lumbar region without neurogenic claudication | Non-infectious |
| M48.062 | Spinal stenosis, lumbar region with neurogenic claudication | Non-infectious |
| M48.54XA | Collapsed vertebra, not elsewhere classified, thoracic region, initial encounter for fracture | Non-infectious |
| M48.56XA | Collapsed vertebra, not elsewhere classified, lumbar region, initial encounter for fracture | Non-infectious |
| M51.04 | Intervertebral disc disorders with myelopathy, thoracic region | Non-infectious |
| M51.16 | Intervertebral disc disorders with radiculopathy, lumbar region | Non-infectious |
| M51.17 | Intervertebral disc disorders with radiculopathy, lumbosacral region | Non-infectious |
| M51.26 | Other intervertebral disc displacement, lumbar region | Non-infectious |
| M51.36 | Other intervertebral disc degeneration, lumbar region | Non-infectious |
| M54.31 | Sciatica, right side | Non-infectious |
| M54.41 | Lumbago with sciatica, right side | Non-infectious |
| M60.9 | Myositis, unspecified | Non-infectious |
| M62.82 | Rhabdomyolysis | Non-infectious |
| M62.830 | Muscle spasm of back | Non-infectious |
| M65.042 | Abscess of tendon sheath, left hand | bone/joint infections |
| M65.141 | Other infective (teno)synovitis, right hand | bone/joint infections |
| M65.142 | Other infective (teno)synovitis, left hand | bone/joint infections |
| M65.161 | Other infective (teno)synovitis, right knee | bone/joint infections |
| M65.172 | Other infective (teno)synovitis, left ankle and foot | bone/joint infections |
| M65.842 | Other synovitis and tenosynovitis, left hand | bone/joint infections |
| M65.88 | Other synovitis and tenosynovitis, other site | bone/joint infections |
| M65.9 | Synovitis and tenosynovitis, unspecified | bone/joint infections |
| M66.0 | Rupture of popliteal cyst | Non-infectious |
| M70.42 | Prepatellar bursitis, left knee | Non-infectious |
| M71.121 | Other infective bursitis, right elbow | SSTI |
| M71.122 | Other infective bursitis, left elbow | SSTI |
| M71.161 | Other infective bursitis, right knee | SSTI |
| M71.22 | Synovial cyst of popliteal space (Baker), left knee | Non-infectious |
| M71.38 | Other bursal cyst, other site | Non-infectious |
| M72.6 | Necrotizing fasciitis | SSTI |
| M75.02 | Adhesive capsulitis of left shoulder | Non-infectious |
| M79.10 | Myalgia, unspecified site | Non-infectious |
| M79.18 | Myalgia, other site | Non-infectious |
| M79.605 | Pain in left leg | Non-infectious |
| M79.81 | Nontraumatic hematoma of soft tissue | Non-infectious |
| M79.89 | Other specified soft tissue disorders | Non-infectious |
| M79.A12 | Nontraumatic compartment syndrome of left upper extremity | Non-infectious |
| M80.031A | Age-related osteoporosis with current pathological fracture, right forearm, initial encounter for fracture | Non-infectious |
| M80.051A | Age-related osteoporosis with current pathological fracture, right femur, initial encounter for fracture | Non-infectious |
| M80.062A | Age-related osteoporosis with current pathological fracture, left lower leg, initial encounter for fracture | Non-infectious |
| M80.08XA | Age-related osteoporosis with current pathological fracture, vertebra(e), initial encounter for fracture | Non-infectious |
| M80.0AXA | Age-related osteoporosis with current pathological fracture, other site, initial encounter for fracture | Non-infectious |
| M80.821A | Other osteoporosis with current pathological fracture, right humerus, initial encounter for fracture | Non-infectious |
| M80.852A | Other osteoporosis with current pathological fracture, left femur, initial encounter for fracture | Non-infectious |
| M80.88XA | Other osteoporosis with current pathological fracture, vertebra(e), initial encounter for fracture | Non-infectious |
| M80.8AXA | Other osteoporosis with current pathological fracture, other site, initial encounter for fracture | Non-infectious |
| M84.351A | Stress fracture, right femur, initial encounter for fracture | Non-infectious |
| M84.422A | Pathological fracture, left humerus, initial encounter for fracture | Non-infectious |
| M84.451A | Pathological fracture, right femur, initial encounter for fracture | Non-infectious |
| M84.452A | Pathological fracture, left femur, initial encounter for fracture | Non-infectious |
| M84.464A | Pathological fracture, left fibula, initial encounter for fracture | Non-infectious |
| M84.474A | Pathological fracture, right foot, initial encounter for fracture | Non-infectious |
| M84.48XA | Pathological fracture, other site, initial encounter for fracture | Non-infectious |
| M84.512A | Pathological fracture in neoplastic disease, left shoulder, initial encounter for fracture | Non-infectious |
| M84.551A | Pathological fracture in neoplastic disease, right femur, initial encounter for fracture | Non-infectious |
| M84.552A | Pathological fracture in neoplastic disease, left femur, initial encounter for fracture | Non-infectious |
| M84.58XA | Pathological fracture in neoplastic disease, other specified site, initial encounter for fracture | Non-infectious |
| M84.662A | Pathological fracture in other disease, left tibia, initial encounter for fracture | Non-infectious |
| M86.08 | Acute hematogenous osteomyelitis, other sites | bone/joint infections |
| M86.131 | Other acute osteomyelitis, right radius and ulna | bone/joint infections |
| M86.141 | Other acute osteomyelitis, right hand | bone/joint infections |
| M86.171 | Other acute osteomyelitis, right ankle and foot | bone/joint infections |
| M86.172 | Other acute osteomyelitis, left ankle and foot | bone/joint infections |
| M86.18 | Other acute osteomyelitis, other site | bone/joint infections |
| M86.362 | Chronic multifocal osteomyelitis, left tibia and fibula | bone/joint infections |
| M86.452 | Chronic osteomyelitis with draining sinus, left femur | bone/joint infections |
| M86.461 | Chronic osteomyelitis with draining sinus, right tibia and fibula | bone/joint infections |
| M86.462 | Chronic osteomyelitis with draining sinus, left tibia and fibula | bone/joint infections |
| M86.471 | Chronic osteomyelitis with draining sinus, right ankle and foot | bone/joint infections |
| M86.60 | Other chronic osteomyelitis, unspecified site | bone/joint infections |
| M86.641 | Other chronic osteomyelitis, right hand | bone/joint infections |
| M86.651 | Other chronic osteomyelitis, right thigh | bone/joint infections |
| M86.661 | Other chronic osteomyelitis, right tibia and fibula | bone/joint infections |
| M86.662 | Other chronic osteomyelitis, left tibia and fibula | bone/joint infections |
| M86.671 | Other chronic osteomyelitis, right ankle and foot | bone/joint infections |
| M86.68 | Other chronic osteomyelitis, other site | bone/joint infections |
| M86.8X3 | Other osteomyelitis, forearm | bone/joint infections |
| M86.8X7 | Other osteomyelitis, ankle and foot | bone/joint infections |
| M86.8X8 | Other osteomyelitis, other site | bone/joint infections |
| M86.9 | Osteomyelitis, unspecified | bone/joint infections |
| M87.152 | Osteonecrosis due to drugs, left femur | Non-infectious |
| M87.38 | Other secondary osteonecrosis, other site | Non-infectious |
| M87.9 | Osteonecrosis, unspecified | Non-infectious |
| M94.1 | Relapsing polychondritis | Non-infectious |
| M95.2 | Other acquired deformity of head | Non-infectious |
| M96.0 | Pseudarthrosis after fusion or arthrodesis | Non-infectious |
| M96.3 | Postlaminectomy kyphosis | Non-infectious |
| M96.69 | Fracture of other bone following insertion of orthopedic implant, joint prosthesis, or bone plate | Non-infectious |
| M96.840 | Postprocedural hematoma of a musculoskeletal structure following a musculoskeletal system procedure | Non-infectious |
| M96.841 | Postprocedural hematoma of a musculoskeletal structure following other procedure | Non-infectious |
| M96.842 | Postprocedural seroma of a musculoskeletal structure following a musculoskeletal system procedure | Non-infectious |
| M96.843 | Postprocedural seroma of a musculoskeletal structure following other procedure | Non-infectious |
| M97.01XA | Periprosthetic fracture around internal prosthetic right hip joint, initial encounter | Non-infectious |
| M97.02XA | Periprosthetic fracture around internal prosthetic left hip joint, initial encounter | Non-infectious |
| N02.8 | Recurrent and persistent hematuria with other morphologic changes | Non-infectious |
| N02.9 | Recurrent and persistent hematuria with unspecified morphologic changes | Non-infectious |
| N04.9 | Nephrotic syndrome with unspecified morphologic changes | Non-infectious |
| N10 | Acute pyelonephritis | Genitourinary |
| N11.8 | Other chronic tubulo-interstitial nephritis | Genitourinary |
| N12 | Tubulo-interstitial nephritis, not specified as acute or chronic | Genitourinary |
| N13.1 | Hydronephrosis with ureteral stricture, not elsewhere classified | Non-infectious |
| N13.2 | Hydronephrosis with renal and ureteral calculous obstruction | Non-infectious |
| N13.6 | Pyonephrosis | Genitourinary |
| N13.8 | Other obstructive and reflux uropathy | Non-infectious |
| N15.1 | Renal and perinephric abscess | Genitourinary |
| N17.0 | Acute kidney failure with tubular necrosis | Non-infectious |
| N17.8 | Other acute kidney failure | Non-infectious |
| N17.9 | Acute kidney failure, unspecified | Non-infectious |
| N18.6 | End stage renal disease | Non-infectious |
| N20.0 | Calculus of kidney | Non-infectious |
| N20.1 | Calculus of ureter | Non-infectious |
| N20.2 | Calculus of kidney with calculus of ureter | Non-infectious |
| N26.1 | Atrophy of kidney (terminal) | Non-infectious |
| N30.00 | Acute cystitis without hematuria | Genitourinary |
| N30.01 | Acute cystitis with hematuria | Genitourinary |
| N30.10 | Interstitial cystitis (chronic) without hematuria | Non-infectious |
| N30.41 | Irradiation cystitis with hematuria | Non-infectious |
| N30.80 | Other cystitis without hematuria | Genitourinary |
| N30.81 | Other cystitis with hematuria | Genitourinary |
| N30.90 | Cystitis, unspecified without hematuria | Genitourinary |
| N30.91 | Cystitis, unspecified with hematuria | Genitourinary |
| N31.9 | Neuromuscular dysfunction of bladder, unspecified | Non-infectious |
| N32.0 | Bladder-neck obstruction | Non-infectious |
| N32.1 | Vesicointestinal fistula | Non-infectious |
| N32.2 | Vesical fistula, not elsewhere classified | Non-infectious |
| N32.89 | Other specified disorders of bladder | Non-infectious |
| N34.0 | Urethral abscess | Genitourinary |
| N34.2 | Other urethritis | Genitourinary |
| N36.0 | Urethral fistula | Non-infectious |
| N39.0 | Urinary tract infection, site not specified | Genitourinary |
| N40.1 | Benign prostatic hyperplasia with lower urinary tract symptoms | Non-infectious |
| N41.0 | Acute prostatitis | Genitourinary |
| N41.1 | Chronic prostatitis | Genitourinary |
| N41.2 | Abscess of prostate | Genitourinary |
| N41.8 | Other inflammatory diseases of prostate | Genitourinary |
| N41.9 | Inflammatory disease of prostate, unspecified | Genitourinary |
| N43.1 | Infected hydrocele | Genitourinary |
| N45.1 | Epididymitis | Genitourinary |
| N45.3 | Epididymo-orchitis | Genitourinary |
| N49.1 | Inflammatory disorders of spermatic cord, tunica vaginalis and vas deferens | Genitourinary |
| N49.2 | Inflammatory disorders of scrotum | Genitourinary |
| N49.3 | Fournier gangrene | Genitourinary |
| N50.1 | Vascular disorders of male genital organs | Non-infectious |
| N61.0 | Mastitis without abscess | SSTI |
| N61.1 | Abscess of the breast and nipple | SSTI |
| N61.21 | Granulomatous mastitis, right breast | Non-infectious |
| N62 | Hypertrophy of breast | Non-infectious |
| N65.0 | Deformity of reconstructed breast | Non-infectious |
| N70.03 | Acute salpingitis and oophoritis | Genitourinary |
| N70.11 | Chronic salpingitis | Genitourinary |
| N70.13 | Chronic salpingitis and oophoritis | Genitourinary |
| N70.91 | Salpingitis, unspecified | Genitourinary |
| N70.93 | Salpingitis and oophoritis, unspecified | Genitourinary |
| N71.9 | Inflammatory disease of uterus, unspecified | Non-infectious |
| N72 | Inflammatory disease of cervix uteri | Non-infectious |
| N73.0 | Acute parametritis and pelvic cellulitis | Genitourinary |
| N73.9 | Female pelvic inflammatory disease, unspecified | Genitourinary |
| N75.1 | Abscess of Bartholin's gland | Genitourinary |
| N76.0 | Acute vaginitis | Genitourinary |
| N76.2 | Acute vulvitis | Genitourinary |
| N76.4 | Abscess of vulva | Genitourinary |
| N80.1 | Endometriosis of ovary | Non-infectious |
| N80.121 | Deep endometriosis of right ovary | Non-infectious |
| N80.9 | Endometriosis, unspecified | Non-infectious |
| N81.9 | Female genital prolapse, unspecified | Non-infectious |
| N82.0 | Vesicovaginal fistula | Non-infectious |
| N82.3 | Fistula of vagina to large intestine | Non-infectious |
| N82.4 | Other female intestinal-genital tract fistulae | Non-infectious |
| N83.292 | Other ovarian cyst, left side | Non-infectious |
| N83.8 | Other noninflammatory disorders of ovary, fallopian tube and broad ligament | Non-infectious |
| N99.520 | Hemorrhage of incontinent external stoma of urinary tract | Non-infectious |
| N99.521 | Infection of incontinent external stoma of urinary tract | Genitourinary |
| N99.528 | Other complication of incontinent external stoma of urinary tract | Non-infectious |
| N99.71 | Accidental puncture and laceration of a Genitourinary system organ or structure during a Genitourinary system procedure | Non-infectious |
| N99.72 | Accidental puncture and laceration of a Genitourinary system organ or structure during other procedure | Non-infectious |
| N99.81 | Other intraoperative complications of Genitourinary system | Non-infectious |
| N99.820 | Postprocedural hemorrhage of a Genitourinary system organ or structure following a Genitourinary system procedure | Non-infectious |
| N99.840 | Postprocedural hematoma of a Genitourinary system organ or structure following a Genitourinary system procedure | Non-infectious |
| N99.89 | Other postprocedural complications and disorders of Genitourinary system | Non-infectious |
| O03.37 | Sepsis following incomplete spontaneous abortion | Genitourinary |
| O03.5 | Genital tract and pelvic infection following complete or unspecified spontaneous abortion | Genitourinary |
| O03.87 | Sepsis following complete or unspecified spontaneous abortion | Genitourinary |
| O23.01 | Infections of kidney in pregnancy, first trimester | Genitourinary |
| O23.02 | Infections of kidney in pregnancy, second trimester | Genitourinary |
| O23.41 | Unspecified infection of urinary tract in pregnancy, first trimester | Genitourinary |
| O26.613 | Liver and biliary tract disorders in pregnancy, third trimester | Non-infectious |
| O26.891 | Other specified pregnancy related conditions, first trimester | Non-infectious |
| O42.912 | Preterm premature rupture of membranes, unspecified as to length of time between rupture and onset of labor, second trimester | Non-infectious |
| O44.02 | Complete placenta previa nos or without hemorrhage, second trimester | Non-infectious |
| O72.1 | Other immediate postpartum hemorrhage | Non-infectious |
| O85 | Puerperal sepsis | Genitourinary |
| O86.01 | Infection of obstetric surgical wound, superficial incisional site | SSTI |
| O91.113 | Abscess of breast associated with pregnancy, third trimester | SSTI |
| O91.22 | Nonpurulent mastitis associated with the puerperium | SSTI |
| O91.23 | Nonpurulent mastitis associated with lactation | SSTI |
| O98.512 | Other viral diseases complicating pregnancy, second trimester | Non-infectious |
| O98.811 | Other maternal infectious and parasitic diseases complicating pregnancy, first trimester | Other |
| O98.812 | Other maternal infectious and parasitic diseases complicating pregnancy, second trimester | Other |
| O98.813 | Other maternal infectious and parasitic diseases complicating pregnancy, third trimester | Other |
| O99.352 | Diseases of the nervous system complicating pregnancy, second trimester | Non-infectious |
| O99.63 | Diseases of the digestive system complicating the puerperium | Non-infectious |
| O99.711 | Diseases of the skin and subcutaneous tissue complicating pregnancy, first trimester | Non-infectious |
| O99.712 | Diseases of the skin and subcutaneous tissue complicating pregnancy, second trimester | Non-infectious |
| O99.891 | Other specified diseases and conditions complicating pregnancy | Non-infectious |
| O9A.211 | Injury, poisoning and certain other consequences of external causes complicating pregnancy, first trimester | Non-infectious |
| Q04.8 | Other specified congenital malformations of brain | Non-infectious |
| Q18.0 | Sinus, fistula and cyst of branchial cleft | Non-infectious |
| Q23.0 | Congenital stenosis of aortic valve | Non-infectious |
| Q23.1 | Congenital insufficiency of aortic valve | Non-infectious |
| Q24.4 | Congenital subaortic stenosis | Non-infectious |
| Q24.5 | Malformation of coronary vessels | Non-infectious |
| Q25.43 | Congenital aneurysm of aorta | Non-infectious |
| Q28.3 | Other malformations of cerebral vessels | Non-infectious |
| Q43.0 | Meckel's diverticulum (displaced) (hypertrophic) | Non-infectious |
| Q44.4 | Choledochal cyst | Non-infectious |
| Q44.6 | Cystic disease of liver | Non-infectious |
| Q61.2 | Polycystic kidney, adult type | Non-infectious |
| Q64.4 | Malformation of urachus | Non-infectious |
| Q80.8 | Other congenital ichthyosis | Non-infectious |
| Q80.9 | Congenital ichthyosis, unspecified | Non-infectious |
| Q82.0 | Hereditary lymphedema | Non-infectious |
| Q85.01 | Neurofibromatosis, type 1 | Non-infectious |
| Q85.02 | Neurofibromatosis, type 2 | Non-infectious |
| Q87.40 | Marfan syndrome, unspecified | Non-infectious |
| Q87.410 | Marfan syndrome with aortic dilation | Non-infectious |
| Q87.418 | Marfan syndrome with other cardiovascular manifestations | Non-infectious |
| Q89.2 | Congenital malformations of other endocrine glands | Non-infectious |
| Q89.8 | Other specified congenital malformations | Non-infectious |
| R00.1 | Bradycardia, unspecified | Non-infectious |
| R04.0 | Epistaxis | Non-infectious |
| R04.2 | Hemoptysis | Non-infectious |
| R06.00 | Dyspnea, unspecified | Non-infectious |
| R06.6 | Hiccough | Non-infectious |
| R07.89 | Other chest pain | Non-infectious |
| R10.0 | Acute abdomen | Non-infectious |
| R10.13 | Epigastric pain | Non-infectious |
| R10.2 | Pelvic and perineal pain | Non-infectious |
| R10.30 | Lower abdominal pain, unspecified | Non-infectious |
| R10.32 | Left lower quadrant pain | Non-infectious |
| R10.84 | Generalized abdominal pain | Non-infectious |
| R10.9 | Unspecified abdominal pain | Non-infectious |
| R11.15 | Cyclical vomiting syndrome unrelated to migraine | Non-infectious |
| R11.2 | Nausea with vomiting, unspecified | Non-infectious |
| R12 | Heartburn | Non-infectious |
| R14.3 | Flatulence | Non-infectious |
| R18.8 | Other ascites | Non-infectious |
| R19.00 | Intra-abdominal and pelvic swelling, mass and lump, unspecified site | Non-infectious |
| R20.2 | Paresthesia of skin | Non-infectious |
| R21 | Rash and other nonspecific skin eruption | Non-infectious |
| R22.0 | Localized swelling, mass and lump, head | Non-infectious |
| R26.89 | Other abnormalities of gait and mobility | Non-infectious |
| R31.0 | Gross hematuria | Non-infectious |
| R33.0 | Drug induced retention of urine | Non-infectious |
| R33.9 | Retention of urine, unspecified | Non-infectious |
| R41.0 | Disorientation, unspecified | Non-infectious |
| R44.2 | Other hallucinations | Non-infectious |
| R50.2 | Drug induced fever | Non-infectious |
| R50.82 | Postprocedural fever | Other |
| R50.9 | Fever, unspecified | Other |
| R53.1 | Weakness | Non-infectious |
| R55 | Syncope and collapse | Non-infectious |
| R56.9 | Unspecified convulsions | Non-infectious |
| R57.1 | Hypovolemic shock | Non-infectious |
| R58 | Hemorrhage, not elsewhere classified | Non-infectious |
| R62.7 | Adult failure to thrive | Non-infectious |
| R73.9 | Hyperglycemia, unspecified | Non-infectious |
| R74.01 | Elevation of levels of liver transaminase levels | Non-infectious |
| R78.81 | Bacteremia | Bloodstream |
| R91.1 | Solitary pulmonary nodule | Non-infectious |
| S01.01XA | Laceration without foreign body of scalp, initial encounter | Non-infectious |
| S01.112A | Laceration without foreign body of left eyelid and periocular area, initial encounter | Non-infectious |
| S01.151A | Open bite of right eyelid and periocular area, initial encounter | Non-infectious |
| S01.512A | Laceration without foreign body of oral cavity, initial encounter | Non-infectious |
| S01.81XA | Laceration without foreign body of other part of head, initial encounter | Non-infectious |
| S02.0XXA | Fracture of vault of skull, initial encounter for closed fracture | Non-infectious |
| S02.122A | Fracture of orbital roof, left side, initial encounter for closed fracture | Non-infectious |
| S02.19XA | Other fracture of base of skull, initial encounter for closed fracture | Non-infectious |
| S02.2XXA | Fracture of nasal bones, initial encounter for closed fracture | Non-infectious |
| S02.31XA | Fracture of orbital floor, right side, initial encounter for closed fracture | Non-infectious |
| S02.32XA | Fracture of orbital floor, left side, initial encounter for closed fracture | Non-infectious |
| S02.40CA | Maxillary fracture, right side, initial encounter for closed fracture | Non-infectious |
| S02.413A | LeFort III fracture, initial encounter for closed fracture | Non-infectious |
| S02.601B | Fracture of unspecified part of body of right mandible, initial encounter for open fracture | Non-infectious |
| S02.611A | Fracture of condylar process of right mandible, initial encounter for closed fracture | Non-infectious |
| S02.611B | Fracture of condylar process of right mandible, initial encounter for open fracture | Non-infectious |
| S02.612A | Fracture of condylar process of left mandible, initial encounter for closed fracture | Non-infectious |
| S02.622A | Fracture of subcondylar process of left mandible, initial encounter for closed fracture | Non-infectious |
| S02.641A | Fracture of ramus of right mandible, initial encounter for closed fracture | Non-infectious |
| S02.652A | Fracture of angle of left mandible, initial encounter for closed fracture | Non-infectious |
| S02.66XA | Fracture of symphysis of mandible, initial encounter for closed fracture | Non-infectious |
| S02.66XB | Fracture of symphysis of mandible, initial encounter for open fracture | Non-infectious |
| S02.69XA | Fracture of mandible of other specified site, initial encounter for closed fracture | Non-infectious |
| S02.69XK | Fracture of mandible of other specified site, subsequent encounter for fracture with nonunion | Non-infectious |
| S02.841B | Fracture of lateral orbital wall, right side, initial encounter for open fracture | Non-infectious |
| S06.0X0A | Concussion without loss of consciousness, initial encounter | Non-infectious |
| S06.320A | Contusion and laceration of left cerebrum without loss of consciousness, initial encounter | Non-infectious |
| S06.349A | Traumatic hemorrhage of right cerebrum with loss of consciousness of unspecified duration, initial encounter | Non-infectious |
| S06.361A | Traumatic hemorrhage of cerebrum, unspecified, with loss of consciousness of 30 minutes or less, initial encounter | Non-infectious |
| S06.369A | Traumatic hemorrhage of cerebrum, unspecified, with loss of consciousness of unspecified duration, initial encounter | Non-infectious |
| S06.4X1A | Epidural hemorrhage with loss of consciousness of 30 minutes or less, initial encounter | Non-infectious |
| S06.5X0A | Traumatic subdural hemorrhage without loss of consciousness, initial encounter | Non-infectious |
| S06.5X1A | Traumatic subdural hemorrhage with loss of consciousness of 30 minutes or less, initial encounter | Non-infectious |
| S06.5X5A | Traumatic subdural hemorrhage with loss of consciousness greater than 24 hours with return to pre-existing conscious level, initial encounter | Non-infectious |
| S06.5X9A | Traumatic subdural hemorrhage with loss of consciousness of unspecified duration, initial encounter | Non-infectious |
| S06.5XAA | Traumatic subdural hemorrhage with loss of consciousness status unknown, initial encounter | Non-infectious |
| S06.6X0A | Traumatic subarachnoid hemorrhage without loss of consciousness, initial encounter | Non-infectious |
| S06.6X1A | Traumatic subarachnoid hemorrhage with loss of consciousness of 30 minutes or less, initial encounter | Non-infectious |
| S06.6X4A | Traumatic subarachnoid hemorrhage with loss of consciousness of 6 hours to 24 hours, initial encounter | Non-infectious |
| S06.6X9A | Traumatic subarachnoid hemorrhage with loss of consciousness of unspecified duration, initial encounter | Non-infectious |
| S06.6XAA | Traumatic subarachnoid hemorrhage with loss of consciousness status unknown, initial encounter | Non-infectious |
| S08.0XXA | Avulsion of scalp, initial encounter | Non-infectious |
| S11.011A | Laceration without foreign body of larynx, initial encounter | Non-infectious |
| S11.81XA | Laceration without foreign body of other specified part of neck, initial encounter | Non-infectious |
| S11.91XA | Laceration without foreign body of unspecified part of neck, initial encounter | Non-infectious |
| S12.01XA | Stable burst fracture of first cervical vertebra, initial encounter for closed fracture | Non-infectious |
| S12.110A | Anterior displaced type ii dens fracture, initial encounter for closed fracture | Non-infectious |
| S12.201A | Unspecified nondisplaced fracture of third cervical vertebra, initial encounter for closed fracture | Non-infectious |
| S12.300A | Unspecified displaced fracture of fourth cervical vertebra, initial encounter for closed fracture | Non-infectious |
| S12.400A | Unspecified displaced fracture of fifth cervical vertebra, initial encounter for closed fracture | Non-infectious |
| S14.104A | Unspecified injury at C4 level of cervical spinal cord, initial encounter | Non-infectious |
| S14.123A | Central cord syndrome at C3 level of cervical spinal cord, initial encounter | Non-infectious |
| S14.127A | Central cord syndrome at C7 level of cervical spinal cord, initial encounter | Non-infectious |
| S14.151A | Other incomplete lesion at C1 level of cervical spinal cord, initial encounter | Non-infectious |
| S15.092A | Other specified injury of left carotid artery, initial encounter | Non-infectious |
| S16.1XXA | Strain of muscle, fascia and tendon at neck level, initial encounter | Non-infectious |
| S16.2XXA | Laceration of muscle, fascia and tendon at neck level, initial encounter | Non-infectious |
| S22.049A | Unspecified fracture of fourth thoracic vertebra, initial encounter for closed fracture | Non-infectious |
| S22.050A | Wedge compression fracture of T5-T6 vertebra, initial encounter for closed fracture | Non-infectious |
| S22.079A | Unspecified fracture of t9-t10 vertebra, initial encounter for closed fracture | Non-infectious |
| S22.081A | Stable burst fracture of t11-T12 vertebra, initial encounter for closed fracture | Non-infectious |
| S22.089A | Unspecified fracture of t11-T12 vertebra, initial encounter for closed fracture | Non-infectious |
| S22.21XA | Fracture of manubrium, initial encounter for closed fracture | Non-infectious |
| S22.31XA | Fracture of one rib, right side, initial encounter for closed fracture | Non-infectious |
| S22.32XA | Fracture of one rib, left side, initial encounter for closed fracture | Non-infectious |
| S22.41XA | Multiple fractures of ribs, right side, initial encounter for closed fracture | Non-infectious |
| S22.42XA | Multiple fractures of ribs, left side, initial encounter for closed fracture | Non-infectious |
| S22.43XA | Multiple fractures of ribs, bilateral, initial encounter for closed fracture | Non-infectious |
| S22.5XXA | Flail chest, initial encounter for closed fracture | Non-infectious |
| S27.0XXA | Traumatic pneumothorax, initial encounter | Non-infectious |
| S27.1XXA | Traumatic hemothorax, initial encounter | Non-infectious |
| S27.2XXA | Traumatic hemopneumothorax, initial encounter | Non-infectious |
| S27.321A | Contusion of lung, unilateral, initial encounter | Non-infectious |
| S27.322A | Contusion of lung, bilateral, initial encounter | Non-infectious |
| S30.0XXA | Contusion of lower back and pelvis, initial encounter | Non-infectious |
| S31.31XA | Laceration without foreign body of scrotum and testes, initial encounter | Non-infectious |
| S31.645A | Puncture wound with foreign body of abdominal wall, periumbilic region with penetration into peritoneal cavity, initial encounter | Non-infectious |
| S32.011A | Stable burst fracture of first lumbar vertebra, initial encounter for closed fracture | Non-infectious |
| S32.019A | Unspecified fracture of first lumbar vertebra, initial encounter for closed fracture | Non-infectious |
| S32.039A | Unspecified fracture of third lumbar vertebra, initial encounter for closed fracture | Non-infectious |
| S32.041A | Stable burst fracture of fourth lumbar vertebra, initial encounter for closed fracture | Non-infectious |
| S32.048A | Other fracture of fourth lumbar vertebra, initial encounter for closed fracture | Non-infectious |
| S32.10XA | Unspecified fracture of sacrum, initial encounter for closed fracture | Non-infectious |
| S32.110A | Nondisplaced zone i fracture of sacrum, initial encounter for closed fracture | Non-infectious |
| S32.119A | Unspecified zone i fracture of sacrum, initial encounter for closed fracture | Non-infectious |
| S32.2XXA | Fracture of coccyx, initial encounter for closed fracture | Non-infectious |
| S32.431A | Displaced fracture of anterior column (iliopubic) of right acetabulum, initial encounter for closed fracture | Non-infectious |
| S32.432A | Displaced fracture of anterior column (iliopubic) of left acetabulum, initial encounter for closed fracture | Non-infectious |
| S32.461A | Displaced associated transverse-posterior fracture of right acetabulum, initial encounter for closed fracture | Non-infectious |
| S32.591A | Other specified fracture of right pubis, initial encounter for closed fracture | Non-infectious |
| S32.592A | Other specified fracture of left pubis, initial encounter for closed fracture | Non-infectious |
| S32.601A | Unspecified fracture of right ischium, initial encounter for closed fracture | Non-infectious |
| S36.031A | Moderate laceration of spleen, initial encounter | Non-infectious |
| S36.032A | Major laceration of spleen, initial encounter | Non-infectious |
| S36.039A | Unspecified laceration of spleen, initial encounter | Non-infectious |
| S36.503A | Unspecified injury of sigmoid colon, initial encounter | Abdomen/pelvis |
| S36.533A | Laceration of sigmoid colon, initial encounter | Abdomen/pelvis |
| S36.893A | Laceration of other intra-abdominal organs, initial encounter | Abdomen/pelvis |
| S37.032A | Laceration of left kidney, unspecified degree, initial encounter | Non-infectious |
| S37.061A | Major laceration of right kidney, initial encounter | Non-infectious |
| S37.13XA | Laceration of ureter, initial encounter | Non-infectious |
| S37.29XA | Other injury of bladder, initial encounter | Non-infectious |
| S37.30XA | Unspecified injury of urethra, initial encounter | Non-infectious |
| S37.828A | Other injury of prostate, initial encounter | Non-infectious |
| S39.012A | Strain of muscle, fascia and tendon of lower back, initial encounter | Non-infectious |
| S40.011A | Contusion of right shoulder, initial encounter | Non-infectious |
| S42.001D | Fracture of unspecified part of right clavicle, subsequent encounter for fracture with routine healing | Non-infectious |
| S42.001K | Fracture of unspecified part of right clavicle, subsequent encounter for fracture with nonunion | Non-infectious |
| S42.202P | Unspecified fracture of upper end of left humerus, subsequent encounter for fracture with malunion | Non-infectious |
| S42.212A | Unspecified displaced fracture of surgical neck of left humerus, initial encounter for closed fracture | Non-infectious |
| S42.291A | Other displaced fracture of upper end of right humerus, initial encounter for closed fracture | Non-infectious |
| S42.295A | Other nondisplaced fracture of upper end of left humerus, initial encounter for closed fracture | Non-infectious |
| S42.301A | Unspecified fracture of shaft of humerus, right arm, initial encounter for closed fracture | Non-infectious |
| S51.852A | Open bite of left forearm, initial encounter | Non-infectious |
| S52.552B | Other extraarticular fracture of lower end of left radius, initial encounter for open fracture type I or II | Non-infectious |
| S56.391A | Other injury of extensor or abductor muscles, fascia and tendons of right thumb at forearm level, initial encounter | Non-infectious |
| S60.562A | Insect bite (nonvenomous) of left hand, initial encounter | Non-infectious |
| S61.051A | Open bite of right thumb without damage to nail, initial encounter | Non-infectious |
| S61.200A | Unspecified open wound of right index finger without damage to nail, initial encounter | Non-infectious |
| S61.205A | Unspecified open wound of left ring finger without damage to nail, initial encounter | Non-infectious |
| S61.235A | Puncture wound without foreign body of left ring finger without damage to nail, initial encounter | Non-infectious |
| S61.253A | Open bite of left middle finger without damage to nail, initial encounter | Non-infectious |
| S61.401A | Unspecified open wound of right hand, initial encounter | Non-infectious |
| S61.412A | Laceration without foreign body of left hand, initial encounter | Non-infectious |
| S61.441A | Puncture wound with foreign body of right hand, initial encounter | Non-infectious |
| S61.442A | Puncture wound with foreign body of left hand, initial encounter | Non-infectious |
| S61.451A | Open bite of right hand, initial encounter | Non-infectious |
| S62.392B | Other fracture of third metacarpal bone, right hand, initial encounter for open fracture | Non-infectious |
| S62.632A | Displaced fracture of distal phalanx of right middle finger, initial encounter for closed fracture | Non-infectious |
| S62.635B | Displaced fracture of distal phalanx of left ring finger, initial encounter for open fracture | Non-infectious |
| S66.125A | Laceration of flexor muscle, fascia and tendon of left ring finger at wrist and hand level, initial encounter | Non-infectious |
| S66.821A | Laceration of other specified muscles, fascia and tendons at wrist and hand level, right hand, initial encounter | Non-infectious |
| S68.012A | Complete traumatic metacarpophalangeal amputation of left thumb, initial encounter | Non-infectious |
| S68.614A | Complete traumatic transphalangeal amputation of right ring finger, initial encounter | Non-infectious |
| S70.01XA | Contusion of right hip, initial encounter | Non-infectious |
| S70.12XA | Contusion of left thigh, initial encounter | Non-infectious |
| S72.001A | Fracture of unspecified part of neck of right femur, initial encounter for closed fracture | Non-infectious |
| S72.002A | Fracture of unspecified part of neck of left femur, initial encounter for closed fracture | Non-infectious |
| S72.002K | Fracture of unspecified part of neck of left femur, subsequent encounter for closed fracture with nonunion | Non-infectious |
| S72.011A | Unspecified intracapsular fracture of right femur, initial encounter for closed fracture | Non-infectious |
| S72.141A | Displaced intertrochanteric fracture of right femur, initial encounter for closed fracture | Non-infectious |
| S72.142A | Displaced intertrochanteric fracture of left femur, initial encounter for closed fracture | Non-infectious |
| S72.402A | Unspecified fracture of lower end of left femur, initial encounter for closed fracture | Non-infectious |
| S72.451A | Displaced supracondylar fracture without intracondylar extension of lower end of right femur, initial encounter for closed fracture | Non-infectious |
| S72.492A | Other fracture of lower end of left femur, initial encounter for closed fracture | Non-infectious |
| S72.91XA | Unspecified fracture of right femur, initial encounter for closed fracture | Non-infectious |
| S76.811A | Strain of other specified muscles, fascia and tendons at thigh level, right thigh, initial encounter | Non-infectious |
| S81.802A | Unspecified open wound, left lower leg, initial encounter | Non-infectious |
| S81.811A | Laceration without foreign body, right lower leg, initial encounter | Non-infectious |
| S81.812A | Laceration without foreign body, left lower leg, initial encounter | Non-infectious |
| S82.002A | Unspecified fracture of left patella, initial encounter for closed fracture | Non-infectious |
| S82.032A | Displaced transverse fracture of left patella, initial encounter for closed fracture | Non-infectious |
| S82.141A | Displaced bicondylar fracture of right tibia, initial encounter for closed fracture | Non-infectious |
| S82.142A | Displaced bicondylar fracture of left tibia, initial encounter for closed fracture | Non-infectious |
| S82.191A | Other fracture of upper end of right tibia, initial encounter for closed fracture | Non-infectious |
| S82.202N | Unspecified fracture of shaft of left tibia, subsequent encounter for open fracture type IIIA, IIIB, or IIIC with nonunion | Non-infectious |
| S82.462C | Displaced segmental fracture of shaft of left fibula, initial encounter for open fracture type IIIA, IIIB, or IIIC | Non-infectious |
| S82.61XA | Displaced fracture of lateral malleolus of right fibula, initial encounter for closed fracture | Non-infectious |
| S82.62XB | Displaced fracture of lateral malleolus of left fibula, initial encounter for open fracture type I or II | Non-infectious |
| S82.842A | Displaced bimalleolar fracture of left lower leg, initial encounter for closed fracture | Non-infectious |
| S82.852A | Displaced trimalleolar fracture of left lower leg, initial encounter for closed fracture | Non-infectious |
| S86.012A | Strain of left Achilles tendon, initial encounter | Non-infectious |
| S92.322A | Displaced fracture of second metatarsal bone, left foot, initial encounter for closed fracture | Non-infectious |
| T16.1XXA | Foreign body in right ear, initial encounter | Non-infectious |
| T17.228A | Food in pharynx causing other injury, initial encounter | Non-infectious |
| T17.590A | Other foreign object in bronchus causing asphyxiation, initial encounter | Non-infectious |
| T17.828A | Food in other parts of respiratory tract causing other injury, initial encounter | Non-infectious |
| T18.128A | Food in esophagus causing other injury, initial encounter | Non-infectious |
| T18.8XXA | Foreign body in other parts of alimentary tract, initial encounter | Non-infectious |
| T38.3X1A | Poisoning by insulin and oral hypoglycemic (antidiabetic) drugs, accidental (unintentional), initial encounter | Non-infectious |
| T39.1X1A | Poisoning by 4-aminophenol derivatives, accidental (unintentional), initial encounter | Non-infectious |
| T39.391A | Poisoning by other nonsteroidal anti-inflammatory drugs (nsaid), accidental (unintentional), initial encounter | Non-infectious |
| T40.2X1A | Poisoning by other opioids, accidental (unintentional), initial encounter | Non-infectious |
| T40.411A | Poisoning by fentanyl or fentanyl analogs, accidental (unintentional), initial encounter | Non-infectious |
| T40.412A | Poisoning by fentanyl or fentanyl analogs, intentional self-harm, initial encounter | Non-infectious |
| T40.711A | Poisoning by cannabis, accidental (unintentional), initial encounter | Non-infectious |
| T42.4X1A | Poisoning by benzodiazepines, accidental (unintentional), initial encounter | Non-infectious |
| T42.4X2A | Poisoning by benzodiazepines, intentional self-harm, initial encounter | Non-infectious |
| T42.6X1A | Poisoning by other antiepileptic and sedative-hypnotic drugs, accidental (unintentional), initial encounter | Non-infectious |
| T43.621A | Poisoning by amphetamines, accidental (unintentional), initial encounter | Non-infectious |
| T44.3X1A | Poisoning by other parasympatholytics (anticholinergics and antimuscarinics) and spasmolytics, accidental (unintentional), initial encounter | Non-infectious |
| T46.0X1A | Poisoning by cardiac-stimulant glycosides and drugs of similar action, accidental (unintentional), initial encounter | Non-infectious |
| T47.1X1A | Poisoning by other antacids and anti-gastric-secretion drugs, accidental (unintentional), initial encounter | Non-infectious |
| T51.2X1A | Toxic effect of 2-propanol, accidental (unintentional), initial encounter | Non-infectious |
| T59.811A | Toxic effect of smoke, accidental (unintentional), initial encounter | Non-infectious |
| T65.891A | Toxic effect of other specified substances, accidental (unintentional), initial encounter | Non-infectious |
| T66.XXXA | Radiation sickness, unspecified, initial encounter | Non-infectious |
| T74.21XA | Adult sexual abuse, confirmed, initial encounter | Non-infectious |
| T78.3XXA | Angioneurotic edema, initial encounter | Non-infectious |
| T79.6XXA | Traumatic ischemia of muscle, initial encounter | Non-infectious |
| T79.7XXA | Traumatic subcutaneous emphysema, initial encounter | Non-infectious |
| T79.A12A | Traumatic compartment syndrome of left upper extremity, initial encounter | Non-infectious |
| T80.211A | Bloodstream infection due to central venous catheter, initial encounter | Bloodstream |
| T80.212A | Local infection due to central venous catheter, initial encounter | SSTI |
| T80.219A | Unspecified infection due to central venous catheter, initial encounter | Bloodstream |
| T80.29XA | Infection following other infusion, transfusion and therapeutic injection, initial encounter | Other |
| T80.82XA | Complication of immune effector cellular therapy, initial encounter | Non-infectious |
| T80.89XA | Other complications following infusion, transfusion and therapeutic injection, initial encounter | Non-infectious |
| T81.30XA | Disruption of wound, unspecified, initial encounter | Other |
| T81.31XA | Disruption of external operation (surgical) wound, not elsewhere classified, initial encounter | Non-infectious |
| T81.32XA | Disruption of internal operation (surgical) wound, not elsewhere classified, initial encounter | Non-infectious |
| T81.40XA | Infection following a procedure, unspecified, initial encounter | Other |
| T81.41XA | Infection following a procedure, superficial incisional surgical site, initial encounter | SSTI |
| T81.42XA | Infection following a procedure, deep incisional surgical site, initial encounter | Other |
| T81.43XA | Infection following a procedure, organ and space surgical site, initial encounter | SSTI |
| T81.44XA | Sepsis following a procedure, initial encounter | Sepsis |
| T81.49XA | Infection following a procedure, other surgical site, initial encounter | SSTI |
| T81.590A | Other complications of foreign body accidentally left in body following surgical operation, initial encounter | Non-infectious |
| T81.718A | Complication of other artery following a procedure, not elsewhere classified, initial encounter | Non-infectious |
| T81.72XA | Complication of vein following a procedure, not elsewhere classified, initial encounter | Non-infectious |
| T81.82XA | Emphysema (subcutaneous) resulting from a procedure, initial encounter | Non-infectious |
| T81.83XA | Persistent postprocedural fistula, initial encounter | Non-infectious |
| T81.89XA | Other complications of procedures, not elsewhere classified, initial encounter | Non-infectious |
| T82.03XA | Leakage of heart valve prosthesis, initial encounter | Non-infectious |
| T82.110A | Breakdown (mechanical) of cardiac electrode, initial encounter | Non-infectious |
| T82.111A | Breakdown (mechanical) of cardiac pulse generator (battery), initial encounter | Non-infectious |
| T82.228A | Other mechanical complication of biological heart valve graft, initial encounter | Non-infectious |
| T82.310A | Breakdown (mechanical) of aortic (bifurcation) graft (replacement), initial encounter | Non-infectious |
| T82.320A | Displacement of aortic (bifurcation) graft (replacement), initial encounter | Non-infectious |
| T82.330A | Leakage of aortic (bifurcation) graft (replacement), initial encounter | Non-infectious |
| T82.49XA | Other complication of vascular dialysis catheter, initial encounter | Non-infectious |
| T82.528A | Displacement of other cardiac and vascular devices and implants, initial encounter | Non-infectious |
| T82.6XXA | Infection and inflammatory reaction due to cardiac valve prosthesis, initial encounter | Endocarditis |
| T82.7XXA | Infection and inflammatory reaction due to other cardiac and vascular devices, implants and grafts, initial encounter | Bloodstream |
| T82.838A | Hemorrhage due to vascular prosthetic devices, implants and grafts, initial encounter | Non-infectious |
| T82.855A | Stenosis of coronary artery stent, initial encounter | Non-infectious |
| T82.856A | Stenosis of peripheral vascular stent, initial encounter | Non-infectious |
| T82.857A | Stenosis of other cardiac prosthetic devices, implants and grafts, initial encounter | Non-infectious |
| T82.858A | Stenosis of other vascular prosthetic devices, implants and grafts, initial encounter | Non-infectious |
| T82.868A | Thrombosis due to vascular prosthetic devices, implants and grafts, initial encounter | Non-infectious |
| T82.897A | Other specified complication of cardiac prosthetic devices, implants and grafts, initial encounter | Non-infectious |
| T82.898A | Other specified complication of vascular prosthetic devices, implants and grafts, initial encounter | Non-infectious |
| T83.020A | Displacement of cystostomy catheter, initial encounter | Non-infectious |
| T83.022A | Displacement of nephrostomy catheter, initial encounter | Non-infectious |
| T83.028A | Displacement of other urinary catheter, initial encounter | Non-infectious |
| T83.031A | Leakage of indwelling urethral catheter, initial encounter | Non-infectious |
| T83.091A | Other mechanical complication of indwelling urethral catheter, initial encounter | Non-infectious |
| T83.092A | Other mechanical complication of nephrostomy catheter, initial encounter | Non-infectious |
| T83.098A | Other mechanical complication of other urinary catheter, initial encounter | Non-infectious |
| T83.113A | Breakdown (mechanical) of other urinary stents, initial encounter | Non-infectious |
| T83.122A | Displacement of indwelling ureteral stent, initial encounter | Non-infectious |
| T83.192A | Other mechanical complication of indwelling ureteral stent, initial encounter | Non-infectious |
| T83.193A | Other mechanical complication of other urinary stent, initial encounter | Non-infectious |
| T83.23XA | Leakage of graft of urinary organ, initial encounter | Non-infectious |
| T83.410A | Breakdown (mechanical) of implanted penile prosthesis, initial encounter | Non-infectious |
| T83.510A | Infection and inflammatory reaction due to cystostomy catheter, initial encounter | Genitourinary |
| T83.511A | Infection and inflammatory reaction due to indwelling urethral catheter, initial encounter | Genitourinary |
| T83.512A | Infection and inflammatory reaction due to nephrostomy catheter, initial encounter | Genitourinary |
| T83.518A | Infection and inflammatory reaction due to other urinary catheter, initial encounter | Genitourinary |
| T83.592A | Infection and inflammatory reaction due to indwelling ureteral stent, initial encounter | Genitourinary |
| T83.593A | Infection and inflammatory reaction due to other urinary stents, initial encounter | Genitourinary |
| T83.598A | Infection and inflammatory reaction due to other prosthetic device, implant and graft in urinary system, initial encounter | Genitourinary |
| T83.61XA | Infection and inflammatory reaction due to implanted penile prosthesis, initial encounter | Genitourinary |
| T83.69XA | Infection and inflammatory reaction due to other prosthetic device, implant and graft in genital tract, initial encounter | Genitourinary |
| T83.711A | Erosion of implanted vaginal mesh to surrounding organ or tissue, initial encounter | Non-infectious |
| T83.83XA | Hemorrhage due to Genitourinary prosthetic devices, implants and grafts, initial encounter | Non-infectious |
| T83.89XA | Other specified complication of Genitourinary prosthetic devices, implants and grafts, initial encounter | Non-infectious |
| T84.020A | Dislocation of internal right hip prosthesis, initial encounter | Non-infectious |
| T84.021A | Dislocation of internal left hip prosthesis, initial encounter | Non-infectious |
| T84.022A | Instability of internal right knee prosthesis, initial encounter | Non-infectious |
| T84.030A | Mechanical loosening of internal right hip prosthetic joint, initial encounter | Non-infectious |
| T84.031A | Mechanical loosening of internal left hip prosthetic joint, initial encounter | Non-infectious |
| T84.033A | Mechanical loosening of internal left knee prosthetic joint, initial encounter | Non-infectious |
| T84.038A | Mechanical loosening of other internal prosthetic joint, initial encounter | Non-infectious |
| T84.060A | Wear of articular bearing surface of internal prosthetic right hip joint, initial encounter | Non-infectious |
| T84.061A | Wear of articular bearing surface of internal prosthetic left hip joint, initial encounter | Non-infectious |
| T84.091A | Other mechanical complication of internal left hip prosthesis, initial encounter | Non-infectious |
| T84.093A | Other mechanical complication of internal left knee prosthesis, initial encounter | Non-infectious |
| T84.216A | Breakdown (mechanical) of internal fixation device of vertebrae, initial encounter | Non-infectious |
| T84.498A | Other mechanical complication of other internal orthopedic devices, implants and grafts, initial encounter | Non-infectious |
| T84.51XA | Infection and inflammatory reaction due to internal right hip prosthesis, initial encounter | bone/joint infections |
| T84.52XA | Infection and inflammatory reaction due to internal left hip prosthesis, initial encounter | bone/joint infections |
| T84.53XA | Infection and inflammatory reaction due to internal right knee prosthesis, initial encounter | bone/joint infections |
| T84.54XA | Infection and inflammatory reaction due to internal left knee prosthesis, initial encounter | bone/joint infections |
| T84.59XA | Infection and inflammatory reaction due to other internal joint prosthesis, initial encounter | bone/joint infections |
| T84.610A | Infection and inflammatory reaction due to internal fixation device of right humerus, initial encounter | bone/joint infections |
| T84.611A | Infection and inflammatory reaction due to internal fixation device of left humerus, initial encounter | bone/joint infections |
| T84.620A | Infection and inflammatory reaction due to internal fixation device of right femur, initial encounter | bone/joint infections |
| T84.621A | Infection and inflammatory reaction due to internal fixation device of left femur, initial encounter | bone/joint infections |
| T84.622A | Infection and inflammatory reaction due to internal fixation device of right tibia, initial encounter | bone/joint infections |
| T84.623A | Infection and inflammatory reaction due to internal fixation device of left tibia, initial encounter | bone/joint infections |
| T84.63XA | Infection and inflammatory reaction due to internal fixation device of spine, initial encounter | bone/joint infections |
| T84.69XA | Infection and inflammatory reaction due to internal fixation device of other site, initial encounter | bone/joint infections |
| T84.7XXA | Infection and inflammatory reaction due to other internal orthopedic prosthetic devices, implants and grafts, initial encounter | bone/joint infections |
| T84.84XA | Pain due to internal orthopedic prosthetic devices, implants and grafts, initial encounter | Non-infectious |
| T84.89XA | Other specified complication of internal orthopedic prosthetic devices, implants and grafts, initial encounter | Non-infectious |
| T85.01XA | Breakdown (mechanical) of ventricular intracranial (communicating) shunt, initial encounter | Non-infectious |
| T85.190A | Other mechanical complication of implanted electronic neurostimulator of brain electrode (lead), initial encounter | Non-infectious |
| T85.41XA | Breakdown (mechanical) of breast prosthesis and implant, initial encounter | Non-infectious |
| T85.44XA | Capsular contracture of breast implant, initial encounter | Non-infectious |
| T85.49XA | Other mechanical complication of breast prosthesis and implant, initial encounter | Non-infectious |
| T85.510A | Breakdown (mechanical) of bile duct prosthesis, initial encounter | Non-infectious |
| T85.518A | Breakdown (mechanical) of other gastrointestinal prosthetic devices, implants and grafts, initial encounter | Non-infectious |
| T85.520A | Displacement of bile duct prosthesis, initial encounter | Non-infectious |
| T85.528A | Displacement of other gastrointestinal prosthetic devices, implants and grafts, initial encounter | Non-infectious |
| T85.590A | Other mechanical complication of bile duct prosthesis, initial encounter | Non-infectious |
| T85.618A | Breakdown (mechanical) of other specified internal prosthetic devices, implants and grafts, initial encounter | Non-infectious |
| T85.628A | Displacement of other specified internal prosthetic devices, implants and grafts, initial encounter | Non-infectious |
| T85.638A | Leakage of other specified internal prosthetic devices, implants and grafts, initial encounter | Non-infectious |
| T85.698A | Other mechanical complication of other specified internal prosthetic devices, implants and grafts, initial encounter | Non-infectious |
| T85.71XA | Infection and inflammatory reaction due to peritoneal dialysis catheter, initial encounter | Abdomen/pelvis |
| T85.730A | Infection and inflammatory reaction due to ventricular intracranial (communicating) shunt, initial encounter | CNS/meningitis |
| T85.731A | Infection and inflammatory reaction due to implanted electronic neurostimulator of brain, electrode (lead), initial encounter | CNS/meningitis |
| T85.732A | Infection and inflammatory reaction due to implanted electronic neurostimulator of peripheral nerve, electrode (lead), initial encounter | Other |
| T85.733A | Infection and inflammatory reaction due to implanted electronic neurostimulator of spinal cord, electrode (lead), initial encounter | CNS/meningitis |
| T85.734A | Infection and inflammatory reaction due to implanted electronic neurostimulator, generator, initial encounter | CNS/meningitis |
| T85.738A | Infection and inflammatory reaction due to other nervous system device, implant or graft, initial encounter | CNS/meningitis |
| T85.79XA | Infection and inflammatory reaction due to other internal prosthetic devices, implants and grafts, initial encounter | Other |
| T85.838A | Hemorrhage due to other internal prosthetic devices, implants and grafts, initial encounter | Non-infectious |
| T85.848A | Pain due to other internal prosthetic devices, implants and grafts, initial encounter | Non-infectious |
| T85.858A | Stenosis due to other internal prosthetic devices, implants and grafts, initial encounter | Non-infectious |
| T85.868A | Thrombosis due to other internal prosthetic devices, implants and grafts, initial encounter | Non-infectious |
| T85.898A | Other specified complication of other internal prosthetic devices, implants and grafts, initial encounter | Non-infectious |
| T85.9XXA | Unspecified complication of internal prosthetic device, implant and graft, initial encounter | Non-infectious |
| T86.03 | Bone marrow transplant infection | Other |
| T86.09 | Other complications of bone marrow transplant | Non-infectious |
| T86.11 | Kidney transplant rejection | Non-infectious |
| T86.12 | Kidney transplant failure | Non-infectious |
| T86.13 | Kidney transplant infection | Genitourinary |
| T86.19 | Other complication of kidney transplant | Non-infectious |
| T86.20 | Unspecified complication of heart transplant | Non-infectious |
| T86.21 | Heart transplant rejection | Non-infectious |
| T86.22 | Heart transplant failure | Non-infectious |
| T86.298 | Other complications of heart transplant | Non-infectious |
| T86.33 | Heart-lung transplant infection | Other |
| T86.39 | Other complications of heart-lung transplant | Non-infectious |
| T86.41 | Liver transplant rejection | Non-infectious |
| T86.43 | Liver transplant infection | Abdomen/pelvis |
| T86.49 | Other complications of liver transplant | Non-infectious |
| T86.5 | Complications of stem cell transplant | Non-infectious |
| T86.810 | Lung transplant rejection | Non-infectious |
| T86.812 | Lung transplant infection | Pulmonary |
| T86.818 | Other complications of lung transplant | Non-infectious |
| T86.819 | Unspecified complication of lung transplant | Non-infectious |
| T86.822 | Skin graft (allograft) (autograft) infection | SSTI |
| T87.0X2 | Complications of reattached (part of) left upper extremity | Non-infectious |
| T87.41 | Infection of amputation stump, right upper extremity | SSTI |
| T87.42 | Infection of amputation stump, left upper extremity | SSTI |
| T87.43 | Infection of amputation stump, right lower extremity | SSTI |
| T87.44 | Infection of amputation stump, left lower extremity | SSTI |
| T87.81 | Dehiscence of amputation stump | Non-infectious |
| T87.89 | Other complications of amputation stump | Non-infectious |
| T88.6XXA | Anaphylactic reaction due to adverse effect of correct drug or medicament properly administered, initial encounter | Non-infectious |
| T88.8XXA | Other specified complications of surgical and medical care, not elsewhere classified, initial encounter | Non-infectious |
| U07.1 | COVID-19 | Non-infectious |
| Z31.0 | Encounter for reversal of previous sterilization | Non-infectious |
| Z40.01 | Encounter for prophylactic removal of breast | Non-infectious |
| Z42.1 | Encounter for breast reconstruction following mastectomy | Non-infectious |
| Z42.8 | Encounter for other plastic and reconstructive surgery following medical procedure or healed injury | Non-infectious |
| Z43.2 | Encounter for attention to ileostomy | Non-infectious |
| Z43.3 | Encounter for attention to colostomy | Non-infectious |
| Z43.6 | Encounter for attention to other artificial openings of urinary tract | Non-infectious |
| Z46.6 | Encounter for fitting and adjustment of urinary device | Non-infectious |
| Z47.32 | Aftercare following explantation of hip joint prosthesis | Non-infectious |
| Z47.33 | Aftercare following explantation of knee joint prosthesis | Non-infectious |
| Z48.290 | Encounter for aftercare following bone marrow transplant | Non-infectious |
| Z51.11 | Encounter for antineoplastic chemotherapy | Non-infectious |
| Z51.12 | Encounter for antineoplastic immunotherapy | Non-infectious |
| Z51.5 | Encounter for palliative care | Non-infectious |
| Z52.6 | Liver donor | Non-infectious |
