# Supplementary material for: Unraveling indications for discharge antibiotics: the Devil’s in the details
Source: Antimicrob Steward Healthc Epidemiol. 2025 Sep 22;5(1):e233. doi: 10.1017/ash.2025.10123 (PMC12509150; doi:10.1017/ash.2025.10123)
Supplement: Prasad et al. supplementary material 2 — Prasad et al. supplementary material [file S2732494X2510123Xsup002.docx]

**Supplementary Table 2.** Antibiotics for pulmonary, genitourinary and *C. difficile* infections per institutional guidelines.

| Pulmonary |
| --- |
| Amoxicillin/clavulanate |
| Cefpodoxime |
| Azithromycin |
| Levofloxacin |
| Doxycycline |
| Genitourinary |
| Nitrofurantoin |
| Cephalexin |
| Amoxicillin/clavulanate |
| Trimethoprim/sulfamethoxazole |
| Cefadroxil |
| Ciprofloxacin |
| Levofloxacin |
| *C. difficile* |
| Fidaxomicin |
| Vancomycin |
